# PBRM1-dependent PBAF targeting is required for EMT and metastasis in breast cancer

**DOI:** 10.1126/sciadv.aed8038

**Published:** 2026-07-29

**Authors:** Alisha Dhiman, Mitchell G. Ayers, Guanming Jiao, Jamie L. McCuiston, Aparna B. Shinde, Saeed Salehin Akhand, Juan Jauregui-Lozano, Marco Hadisurya, Elizabeth G. Porter, W. Andy Tao, Vikki M. Weake, Yucheng Zhang, Sagar M. Utturkar, Matthew R. Marunde, Michael K. Wendt, Emily C. Dykhuizen

**Affiliations:** ^1^ Borch Department of Medicinal Chemistry and Molecular Pharmacology, Purdue University, West Lafayette, IN 47907, USA.; ^2^ Purdue Institute for Cancer Research Purdue University, West Lafayette, IN 47907, USA.; ^3^ Department of Basic Medical Sciences, Purdue University, West Lafayette, IN 47907, USA.; ^4^ EpiCypher Inc., Durham NC 27709, USA.; ^5^ Department of Biochemistry, Purdue University, West Lafayette, IN 47907, USA.; ^6^ Rosen Center for Advanced Computing (RCAC), Purdue University, West Lafayette, IN 47907, USA.

## Abstract

SWI/SNF chromatin remodelers are represented by three biochemically distinct subcomplexes, the abundant cBAF and the less abundant PBAF and GBAF. Genetics have identified important roles for PBAF in development and disease; however, relating PBAF-mediated phenotypes to biochemical function in chromatin regulation and gene activation has been challenging. Here, we show that the PBRM1 subunit of PBAF is critical for the completion of TGFβ1-mediated epithelial-mesenchymal transition (EMT) of mammary cells in vitro as well as the metastasis of murine breast cancers in vivo. Using epigenomics to profile different stages of EMT, we find that PBRM1 is necessary for targeting PBAF to inducible promoters marked by H3K14ac. We further find that PBRM1 facilitates DNA accessibility at sites bound by TGFβ1-inducible transcription factors, such as Atf3, for the induction of genes involved in migration, cell survival, and inflammation, providing evidence that PBAF is a vulnerability in late-stage metastatic cancers.

## Teaser

During EMT, PBRM1 targets PBAF to H3K14Ac sites, opening chromatin to activate TGFβ targets and enable breast cancer metastasis.

## INTRODUCTION

Mammalian Switch/Sucrose Non-Fermentable (SWI/SNF) chromatin remodeling complexes (also termed BAF for Brg1/Brm-associated factors) are multisubunit complexes that use the energy of adenosine triphosphate to mobilize nucleosomes and create regions of DNA accessibility. SWI/SNF complexes are highly heterogeneous, with hundreds of different combinations of subunits that facilitate cell type–specific transcription programs (*1*). In addition, all cells contain three biochemically distinct SWI/SNF complexes that can be differentiated by size and abundance: canonical BAF (cBAF), polybromo-associated BAF (PBAF), and, most recently, GLTSCR1/1L-associated BAF (GBAF) (*2*) [also called noncanonical BAF (ncBAF) (*3*, *4*)]. cBAF contains the unique subunits ARID1A/B and DPF1/2/3 and is typically the most heterogeneous and abundant of the three SWI/SNF complexes (*3*). It is also primarily responsible for the largest phenotypic and transcriptional effects in most cells (*5*–*7*). Most of the SWI/SNF-driven chromatin accessibility regulation is mediated through cBAF-driven remodeling at enhancers (*8*–*13*). In contrast, PBAF, with unique subunits PHF10/ARID2/BRD7/PBRM1, and GBAF, with unique subunits GLTSCR1or1L/BRD9, seem to contribute very little to overall accessibility (*5*, *14*). For GBAF, this is likely due to low expression and the lack of SMARCB1, which is critical for nucleosome eviction (*15*, *16*). PBAF, however, has critical interactions between core subunits (*16*, *17*) and the nucleosome (*17*, *18*) and cBAF-comparable remodeling activity in vitro (*19*). The reasons behind PBAF’s relatively minor contribution to accessibility in cells remain poorly understood.

Most functional studies of PBAF focus on the roles of PBRM1 and ARID2 in cancers where they are highly mutated (*20*, *21*). In these cancer backgrounds, the effects of PBAF subunit depletion are variable and context dependent, sometimes failing to display obvious tumor-suppressive phenotypes (*22*). In cell systems where PBAF does have a tumor-suppressive phenotype, the effects are often gain of function, whereby PBAF subunit deletion leads to an increase in the activity of other transcriptional factors. For instance, hypoxia-inducible factor 1 or nuclear factor κB activity is increased in renal cancers with PBRM1 deletion (*23*, *24*), and cBAF and Activator Protein-1 (AP-1) activity is increased in melanoma with ARID2 deletion (*25*). In these contexts, PBAF is important for its ability to repress gene expression (*26*) by reducing cBAF abundance (*25*), by moving nucleosomes to block transcription at sites of DNA damage (*26*, *27*) or by facilitating the binding of repressive transcription factors (TFs), such as RE1-Silencing Transcription factor (REST) (*14*). These studies elucidated how deletion of PBAF facilitates the activation of oncogenic pathways in specific cancer contexts (*23*–*26*); however, how these mechanisms may relate to the biochemical mechanism of PBAF-mediated gene activation is still not understood.

In our previous publication, we found that deletion of PBRM1 in epithelial cells increases proliferation under normal growth conditions but decreases viability of the same cell type upon induction of cell stress (*22*). Here, we report that these same epithelial cells with PBRM1 deletion are unable to survive transforming growth factor–β1 (TGFβ1) treatment and complete epithelial-mesenchymal transition (EMT). We use a multiomic approach to defining the transcriptional role for PBRM1 in gene activation during EMT and the related nucleosome binding and chromatin remodeling changes required for this cell state transition. We further demonstrate that this function is essential for metastasis in a variety of mouse models, implicating PBAF as a potential target in metastatic disease.

## RESULTS

### Pbrm1-depleted cells have reduced capacity to undergo EMT

**Fig. 1.**
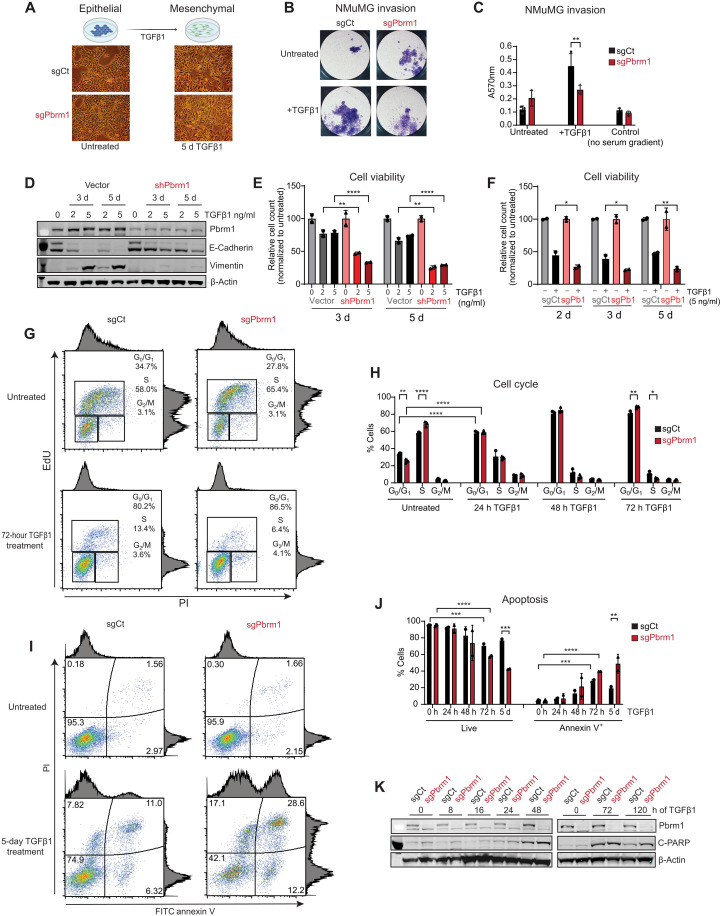
Pbrm1 is important for EMT in NMuMG mouse mammary epithelial cells. (**A**) Schematic of NMuMG treatment with TGFβ1 [created in BioRender. Dykhuizen, E. (2026) https://BioRender.com/gx3lpr0] and associated micrographs (×4 magnification). (**B**) Transwell invasion assay images. (**C**) Quantification of absorbance at 570 nm of solubilized transwells in (B). *n* = 3 replicates. (**D**) Immunoblots of NMuMG whole-cell lysates. β-Actin is used as a loading control. (**E** and **F**) Cell counts of NMuMG cells with shRNA knockdown (E) or sgRNA knockout (F) normalized to untreated cells. Representative graph, *n* = 3 biological replicates. (**G**) Flow cytometry density dot plots of EdU and PI staining in fixed, permeabilized NMuMG cells. The gating strategy for cells in different cell cycle stages (G_0_/G_1_, S, or G_2_/M) is indicated with boxes. (**H**) The percentage of NMuMG cells in different cell cycle stages as quantitated in (G). *n* = 3 biological replicates. (**I**) Flow cytometry density dot plots of annexinV and PI staining in unfixed live NMuMG cells. The gating strategy for the percentage of live (PI^−^)/dead (PI^+^) and apoptotic (annexin V^+^)/nonapoptotic (annexin V^−^) cells is indicated with quadrants. (**J**) The percentage of PI^−^ live cells (left) and annexin V^+^ apoptotic cells (right). Gating was performed as in (I). *n* = 3 biological replicates. (**K**) Immunoblots of whole-cell lysates from NMuMG cells. β-Actin is used as a loading control. Data are represented as mean ± SD. Statistical comparison was done using multiple unpaired *t* tests with Holm-Sidak correction. *P < 0.05, **P < 0.01, ***P < 0.001, and ****P < 0.0001. d, days; h, hours.

EMT confers migration and invasion capabilities to epithelial cells (*28*), facilitating processes like metastasis. In our previous publication (*22*), we found that depletion of *Pbrm1* in epithelial cells resulted in a slight increase in proliferation and a slight reduction in epithelial organization. Further, E-cadherin expression was slightly decreased, leading us to hypothesize that loss of *Pbrm1* facilitates EMT. To test this hypothesis, we treated the normal murine mammary gland (NMuMG) cells with TGFβ1, a cytokine used to induce robust EMT (*28*). Five days of TGFβ1 treatment resulted in a morphology change from tightly packed, cobblestone-like epithelial cells to extended, spindle-like mesenchymal cells (Fig. 1A), which display increased invasion in vitro (Fig. 1, B and C, and fig. S1A). While cells transduced with lentiviral-mediated short hairpin RNA (shRNA) against *Pbrm1* initially appeared to lose their epithelial morphology and gain mesenchymal characteristics (Fig. 1A); contrary to our hypothesis, they did not show accelerated EMT. Instead, *Pbrm1*-depleted cells failed to transition to a mesenchymal state upon addition of TGFβ1, as demonstrated by compromised invasive potential (Fig. 1, B and C, and fig. S1A) and an inability to induce expression of the mesenchymal marker vimentin (Fig. 1D). After removing TGFβ1 for 2 days, E-cadherin levels in cells with sh*Pbrm1* began to rise again, while E-cadherin levels stayed repressed in control cells, substantiating their inability to transition to a mesenchymal state (fig. S1B). This was accompanied by significantly reduced cell viability in sh*Pbrm1* (Fig. 1E) or sg*Pbrm1* (sgPb1) (Fig. 1F) cells treated with TGFβ1. Similarly, cells with sh*Brd7*, a subunit of the PBAF chromatin remodeling complex required for Pbrm1 incorporation and stability within the complex (*3*), displayed a reduction in cell numbers upon TGFβ1 treatment (fig. S1, C to E).

### Pbrm1-depleted epithelial cells exhibit different kinetics of cell cycle and apoptosis under TGFβ1 treatment in vitro

To define whether the reduction in cell numbers in *Pbrm1*-depleted cells with TGFβ1 treatment is due to a reduction of proliferation or an increase in cell death, we evaluated cell cycle and apoptosis. In untreated sg*Pbrm1* cells, a higher percentage of cells were in S phase, which is consistent with the higher growth rate for sg*Pbrm1* cells (Fig. 1, G and H). In normal epithelial cells, TGFβ1 treatment increases the proportion of cells in G_1_, which is required to complete EMT (*29*). After 24 hours of TGFβ1 treatment, both sg*Pbrm1* cells and sgControl (sgCt) cells had reduced proliferation and a similar proportion of cells in G_0_/G_1_. With extended TGFβ1 treatment, however, sgCt cells continued proliferating, while sg*Pbrm1* cells display a near complete exit from the cell cycle, with all cells in G_0_/G_1_ (Fig. 1, G and H, and fig. S1F).

TGFβ1 treatment also induces apoptosis in the subset of NMuMG epithelial cells in G_2_/M until the cells become mesenchymal (*29*). To assess apoptosis in sg*Pbrm1* cells under TGFβ1 treatment, we used cleaved poly(ADP-ribose) polymerase (PARP) and annexin V–propidium iodide (PI) staining. Apoptosis was observed at a similar rate in both groups until 48 hours of treatment (Fig. 1, I and J, and fig. S1G). After 48 hours, there were higher rates of apoptosis in sg*Pbrm1* cells based on both elevated cleaved PARP levels and annexin V staining (Fig. 1, I to K, and fig. S1G). Thus, with extended TGFβ1 treatment (4 to 5 days), the sg*Pbrm1* cells exhibited both a cell cycle block and higher cell death relative to control cells.

### Transcriptional profiling of NMuMG cells with TGFβ1 treatment

**Fig. 2.**
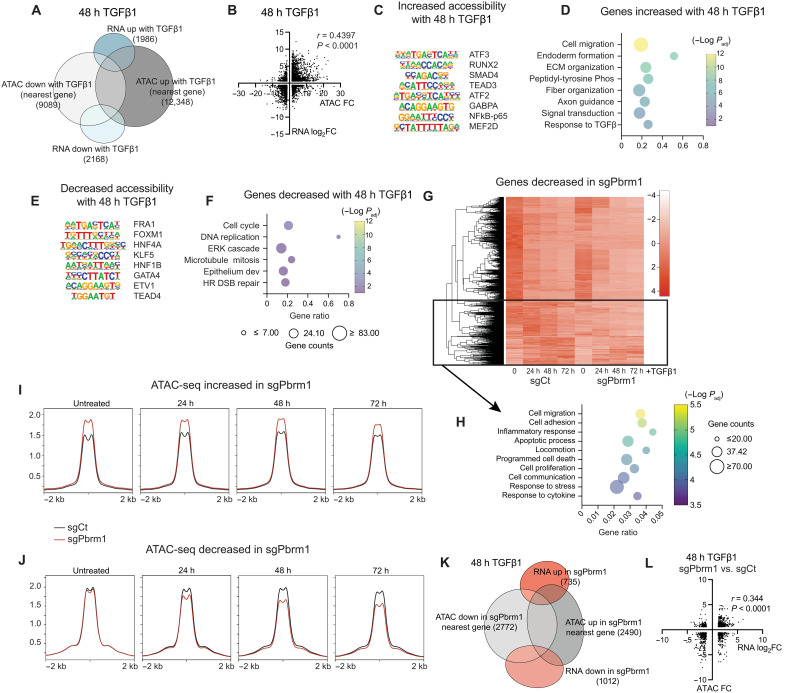
RNA expression and DNA accessibility changes upon TGFβ1-mediated EMT in NMuMG wild-type and Pbrm1 knockout cells. (**A**) Venn diagram showing overlap between DEGs and genes nearest to differentially accessible regions (DARs) in 48-hour TGFβ1-treated cells compared to untreated cells. Total gene numbers are indicated. (**B**) Scatterplot of the log_2_ fold change (FC) of all DEGs from (A) with the FC of all DARs annotated to that gene. Each point represents one gene. (**C**) Motif analysis using Homer on genes with increased expression and accessibility in sgCt cells with 48-hour TGFβ1 treatment. Gene sets were defined using overlap analysis in (A). (**D**) Gene Ontology enrichment analysis was performed on genes with increased expression and accessibility in sgCt cells with 48-hour TGFβ1. Gene sets were defined using overlap analysis in (A). (**E** and **F**) Same as in (C) and (D) but for genes with decreased expression and accessibility. HR DSB, homologous recombination double strand break. (**G**) Heatmap representation of all genes decreased in sg*Pbrm1* relative to sgCt cells in any treatment condition. Genes induced by TGFβ1 in sgCt but not in sg*Pbrm1* cells are subsetted with a box. (**H**) Gene Ontology enrichment analysis was performed on the highlighted subset of genes from (G). (**I** and **J**) Metagene plots of all sites of increased (I) or decreased (J) accessibility in sg*Pbrm1* cells relative to sgCt cells in any treatment condition. Peak summits are aligned at the center. (**K**) Venn diagram showing overlap between DEGs and genes nearest to DARs in sg*Pbrm1* cells relative to sgCt cells following 48-hour TGFβ1 treatment. Total gene numbers are indicated. (**L**) Scatterplot of the log_2_FC of all DEGs from (K) with the FC of all DARs annotated to that gene. Each point represents one gene. ECM, extracellular matrix; ERK, extracellular signal–regulated kinase.

The induction of EMT is progressive, occurring over several days. Previous work has characterized the early changes in gene expression and chromatin accessibility in NMuMG epithelial cells upon TGFβ1 treatment (2 and 12 hours) (*30*). Since *Pbrm1* depletion affects cells during later stages of EMT (Fig. 1, D to K, and fig. S1B), we evaluated gene expression after 24, 48, and 72 hours of TGFβ1 treatment. At each time point, TGFβ1 robustly regulated many genes [between 3133 and 4898 differentially expressed genes (DEGs)] with *P*_adj_ < 0.05, |fold change (FC)| > 1.5. Most of DEGs were common between the time points (fig. S2A) but displayed different kinetics (fig. S2B). To identify how these gene expression changes correlate with changes in chromatin accessibility, we also performed assay for transposase-accessible chromatin (ATAC)–seq. TGFβ1 treatment led to substantial changes in chromatin accessibility at all the time points with ∼15 to 20% of sites completely gained or lost (fig. S2, C and D). To determine whether changes in chromatin accessibility were associated with changes in gene expression, we compared sites of differential accessibility with the changes in gene expression of the nearest gene (Fig. 2A). Overall, the changes in accessibility correlate with changes in gene expression (Fig. 2B and fig. S2E). Sites with increased accessibility and gene expression were enriched for TF motifs for Activating Transcription Factor 3 (ATF3), Runt-related Transcription Factor 2 (RUNX2), Small Mothers Against Decapentaplegic 4 (SMAD4), and TEA Domain Transcription Factor 3 (TEAD3), which are all associated with breast cancer EMT (Fig. 2C) (*31*–*34*); the associated genes were enriched in pathways such as cell migration, extracellular matrix organization, and response to TGFβ1 (Fig. 2D). Sites associated with decreased accessibility and gene expression were enriched for motifs for Fos-Related Antigen 1 (FRA1), Forkhead Box Protein M1 (FOXM1), Hepatocyte Nuclear Factor 4 alpha (HNF4A), and Krueppel-like Factor 5 (KLF5), which are all involved in various aspects of breast epithelium (Fig. 2E) (*35*–*37*); the associated genes were enriched in pathways such as cell cycle, DNA replication, and epithelium development (Fig. 2F). Overall, our RNA sequencing (RNA-seq) and ATAC-seq datasets capture the gene expression and chromatin architecture changes induced by TGFβ1 in NMuMG cells.

### Transcriptional profiling of *Pbrm1* knockout cells under TGFβ1 treatment

To understand how Pbrm1 affects TGFβ1-dependent gene expression, we also performed bulk RNA-seq on sg*Pbrm1* NMuMG cells with increasing TGFβ1 incubation time (fig. S2F). We identified between 1100 and 1800 DEGs in sg*Pbrm1* cells compared to sgCt, with both shared and unique DEGs across the different treatment conditions (fig. S2G). Most TGFβ1-mediated gene expression changes in sgCt were maintained in sg*Pbrm1* cells; however, there was a subset of ∼300 genes that failed to be induced by TGFβ1 in sg*Pbrm1* cells (Fig. 2G, highlighted, and fig. S2H). Consistent with a role for Pbrm1 in facilitating TGFβ1-dependent gene induction, pathway analysis on Pbrm1-dependent genes enriched for processes like cell migration, adhesion, inflammatory response, apoptosis, and response to cytokine (Fig. 2H).

### Differential accessibility of *Pbrm1* knockout cells under TGFβ1 treatment

To determine how Pbrm1 contributes to chromatin accessibility during EMT, we also performed ATAC-seq in the sg*Pbrm1* and sgCt cells (fig. S2I). To identify changes in accessibility with Pbrm1 loss, we performed magnetic-activated cell sorting (MACS) analysis and identified 5000 to 10,000 sites of differential accessibility (*P*_adj_ < 0.05, |FC| > 2) in sg*Pbrm1* cells compared to sgCt cells across the different treatments (fig. S2J). In untreated cells, there were more sites with increased accessibility in sg*Pbrm1* cells than sites with decreased accessibility, as well as slightly higher global accessibility in sg*Pbrm1* cells (fig. S2K), which is consistent with other cell types (*11*, *25*). In TGFβ1-treated cells, however, there were more sites with decreased accessibility in the sg*Pbrm1* cells (fig. S2J). The metagene analysis indicates that the magnitude of sites with increased accessibility in sg*Pbrm1* cells is consistent across treatments (Fig. 2I), while the magnitude of decreased accessibility is much greater with TGFβ1 treatment (Fig. 2J). At 48 hours of TGFβ1, there is an overlap between Pbrm1-dependent gene expression and accessibility (Fig. 2K) as well as a significant positive correlation between DEGs and changes in accessibility at neighboring sites (*r* = 0.344, *P* < 0.0001) (Fig. 2L). Together, these analyses demonstrate that Pbrm1 is necessary for TGFβ1-dependent gene expression and chromatin accessibility.

### Genome occupancy of PBAF in NMuMG cells under TGFβ1 treatment

**Fig. 3.**
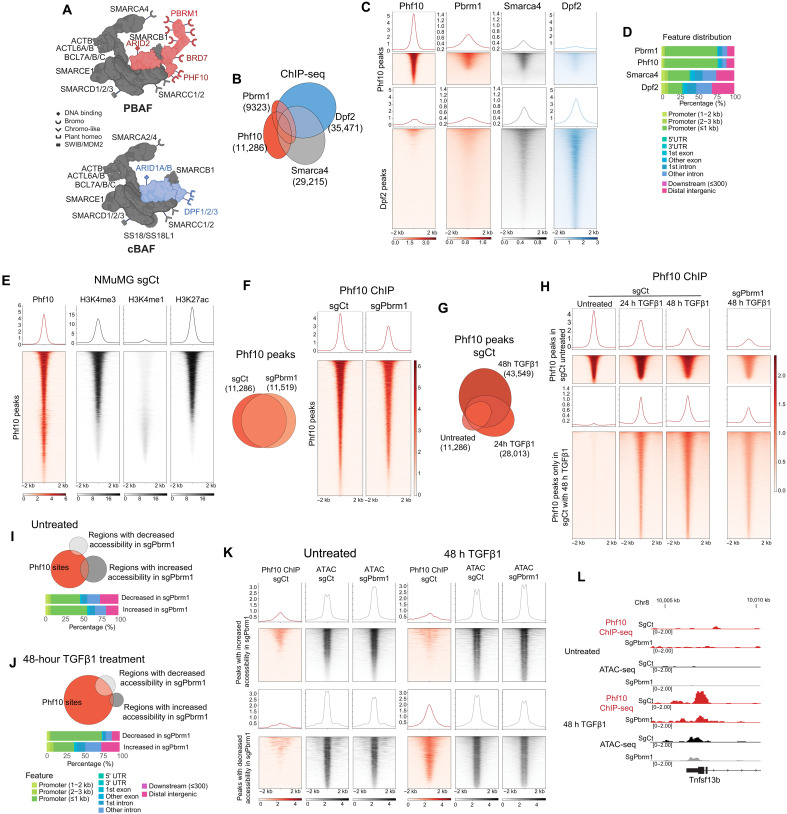
Upon TGFβ1 treatment, PBAF is recruited to new genomic sites to increase DNA accessibility. (**A**) Schematic of PBAF and cBAF complexes. Shared subunits are shown in gray, PBAF-specific subunits in red, and cBAF-specific subunits in blue; reader domains are indicated. Created in BioRender. Dykhuizen, E. (2026) https://BioRender.com/iuu24uw. (**B**) Venn diagram showing overlap of Phf10 and Smarca4 ChIP-seq peaks in sgCt NMuMG cells with Pbrm1 and Dpf2 ChIP-seq peaks from NMuMG cells (GSE249211). Total peak numbers are indicated. (**C**) Metagene plots and heatmaps of Phf10, Pbrm1, Smarca4, and Dpf2 ChIP-seq enrichment at Phf10 and Dpf2 sites defined in (B). (**D**) Genomic feature distribution of ChIP-seq peaks from (B). (**E**) Metagene plots and heatmaps showing enrichment of Phf10, H3K4me3, H3K4me1, and H3K27ac at Phf10 ChIP-seq sites from (B). (**F**) Left: Venn diagram of Phf10 ChIP-seq peaks in sgCt and sg*Pbrm1* cells, with total peak numbers indicated. Right: Metagene plots and heatmaps of Phf10 enrichment at sgCt Phf10 sites; peak summits are centered. (**G**) Venn diagram of Phf10 ChIP-seq peaks in untreated and TGFβ1-treated sgCt cells, with total peak numbers indicated. (**H**) Metagene plots and heatmaps of Phf10 enrichment at untreated Phf10 sites (top) and the TGFβ1-induced Phf10 sites absent in untreated cells (bottom); peak summits are centered. Venn diagrams showing overlap of Phf10 peaks with regions of decreased chromatin accessibility in sg*Pbrm1* versus sgCt cells under untreated (**I**) or 48-hour TGFβ1-treated (**J**) conditions. Genomic feature distributions of differential accessibility sites are shown below. (**K**) Metagene plots and heatmaps of Phf10 ChIP-seq and ATAC-seq enrichment at sites of differential accessibility in sg*Pbrm1* cells under untreated (left) and 48-hour TGFβ1-treated (right) conditions. (**L**) Genome tracks of Phf10 ChIP-seq and ATAC-seq signal at the *Tnfsf13b* locus. 5′UTR, 5′ untranslated region.

To define which of the TGFβ1-regulated genes are direct targets of Pbrm1, we performed chromatin immunoprecipitation sequencing (ChIP-seq) for Phf10, a subunit of PBAF with high-quality ChIP antibodies, along with Smarca4, the adenosine triphosphatase subunit present in all BAF subcomplexes (Fig. 3A). Using MACS, we identified 11,286 robust peaks and good agreement between replicates (fig. S3A). We compared these sites with published NMuMG ChIP-seq for Pbrm1 and Dpf2, a subunit unique to cBAF complexes (*38*). As expected, we observed good overlap between Phf10 peaks and Pbrm1 peaks (∼50% of Pbrm1 sites), moderate overlap with Smarca4 (∼25% of Smarca4 sites), and poor overlap with Dpf2 (∼15% of Dpf2 sites) (Fig. 3B). In accordance with their presence in different complexes, Phf10 sites displayed low Dpf2 enrichment and vice versa, while Smarca4 was enriched at both subsets of sites (Fig. 3C). Similar to data from other cell lines (*25*, *39*), Phf10 and Pbrm1 (PBAF) were primarily localized at promoters, Dpf2 (cBAF) was more enriched at intronic and intergenic sites, and Smarca4 was found at both (Fig. 3D). Confirming the enrichment of PBAF at promoters, Phf10 sites showed high enrichment of histone 3 (H3) K4me3 and H3K27ac and low enrichment of H3K4me1 histone modifications (Fig. 3E).

Pbrm1 is the last subunit to be incorporated into PBAF (*3*), and hence, PBAF complexes can form and exist in the absence of Pbrm1 (*40*). Confirming this, immunoprecipitation–mass spectrometry (IP-MS) of PBAF subunit Phf10 in sg*Pbrm1* cells identified all PBAF subunits in both untreated and TGFβ1-treated cells (fig. S3B). Therefore, we used Phf10 ChIP-seq to determine how loss of Pbrm1 affects PBAF chromatin binding. There was a high overlap between Phf10 peaks from sgCt and sg*Pbrm1* cells (Fig. 3F), indicating that PBAF retains binding to chromatin in the absence of Pbrm1; however, there was a decrease in overall Phf10 enrichment in sg*Pbrm1* cells, which was confirmed by quantitative polymerase chain reaction (qPCR) in independent replicates (Fig. 3F and fig. S3C). This is in agreement with our previous findings that Pbrm1 loss decreases, but does not abrogate, PBAF affinity to chromatin (*39*, *40*).

To identify how PBAF localization changes during EMT, we next performed Phf10 ChIP-seq at 24 and 48 hours of TGFβ1 treatment. Most of the Phf10 sites identified in untreated NMuMG cells were maintained across conditions; however, many additional peaks appeared with 24 and 48 hours of TGFβ1 treatment (Fig. 3G and fig. S3D), all of which were reduced in sg*Pbrm1* cells (Fig. 3H and fig. S3, E and F). These findings indicate that Pbrm1 plays a critical role in PBAF binding and redistribution to TGFβ1-inducible sites.

### Phf10 binding only correlates with Pbrm1-dependent accessibility with TGFβ1 treatment

Next, we evaluated how PBAF binding relates to changes in accessibility in sg*Pbrm1* cells. In untreated cells, Phf10 is not highly enriched at sites with differential accessibility in sg*Pbrm1* cells (Fig. 3I). With 48 hours of TGFβ1 treatment, however, Phf10 peaks do overlap with (Fig. 3J) and are enriched at (Fig. 3K) sites of decreased accessibility in sg*Pbrm1* cells. Collectively, these data suggest that TGFβ1-inducible promoters such as *Tnfsf13b*, a cytokine associated with immune escape and poor prognosis in cancer (*41*), require Pbrm1/PBAF for maximal increased accessibility (Fig. 3L), while constitutive, active promoters do not require Pbrm1 for maintaining accessibility (fig. S3, G and H).

### PBAF cooperates with inducible TFs during EMT

**Fig. 4.**
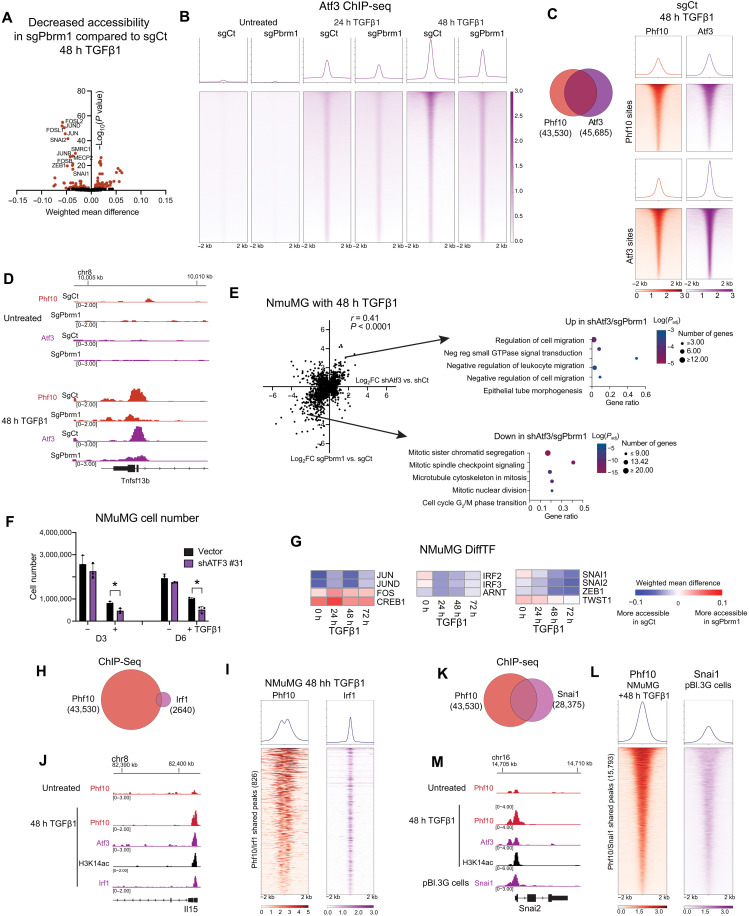
Upon TGFβ1 treatment, Pbrm1 facilitates the binding of inducible TFs. (**A**) Volcano plot of TF consensus motifs enriched at regions of differential chromatin accessibility in sg*Pbrm1* versus sgCt cells following 48-hour TGFβ1 treatment, identified using DiffTF. (**B**) Metagene plots and heatmaps of Atf3 enrichment at Atf3 ChIP-seq sites compiled from all conditions. (**C**) Left: Venn diagram of Phf10 and Atf3 ChIP-seq peaks in sgCt cells treated with TGFβ1 for 48 hours. Right: Metagene plots and heatmaps of Phf10 (top) and Atf3 (bottom) enrichment at their respective binding sites. (**D**) Genome tracks of Phf10 and Atf3 ChIP-seq signal at the *Tnfsf13b* locus. (**E**) Scatterplot comparing log_2_FC of DEGs in sg*Pbrm1* versus sgCt cells and shAtf3 versus shScr cells following 48-hour TGFβ1 treatment. Pearson correlation was calculated using all DEGs. Gene Ontology enrichment for genes up-regulated or down-regulated in both conditions. (**F**) Cell counts of control and shAtf3 cells ± TGFβ1 treatment seeded at 0.9 million and counted on days 3 (D3) and 6 (D6). *n* = 3 biological replicates; mean ± SD. (**G**) Heatmap of differential accessibility for consensus TF motifs in sg*Pbrm1* versus sgCt cells determined by DiffTF. (**H** and **I**) Overlap of Phf10 ChIP-seq peaks with Irf1 ChIP-seq peaks (GSE141501) from NMuMG cells with 48-hour TGFβ1. Metagene plots and heatmaps are for shared sites. (**J**) Genome tracks of ChIP-Seq enrichment at the *Il15* locus. (**K** and **L**) Overlap of Phf10 ChIP-seq peaks from NMuMG cells with 48-hour TGFβ1 with Snai1 ChIP-seq peaks from pBI3G mouse mesenchymal breast cancer cells (GSE61198). Metagene plots and heatmaps are for shared sites. (**M**) Genome tracks of ChIP-seq enrichment at the *Snai2* locus.*P < 0.05, **P < 0.01, ***P < 0.001, and ****P < 0.0001. GTPase, guanosine triphosphatase.

To identify potential TFs that require PBAF for binding, we performed Quantification of Differential Transcription Factor Activity (DiffTF) (*42*) analysis on our ATAC-seq data. Regions that required Pbrm1 for increased accessibility upon TGFβ1 treatment were enriched for AP-1 motifs (Jun, Junb, Jund, Fosb, Fosl1, and Fosl2), EMT TFs (Snai1, Snai2, and Zeb1), SWI/SNF (Smarcc1), and MeCP2 (methyl CpG binding protein 2) (Fig. 4A). cBAF has been demonstrated to cooperate with the AP-1 family of TFs at enhancers (*43*, *44*); however, this has not been demonstrated for PBAF at promoters. To elucidate the AP-1 subunits specifically important for TGFβ1-inducible gene expression, we used our RNA-seq datasets to identify which AP-1 subunit genes are induced by TGFβ1. The expressions of *Jun*, *Junb*, *Fosl2*, *Fosb*, and *Atf3* were all substantially increased upon TGFβ1 treatment (fig. S4A). We next performed ChIP-seq for these five TFs and obtained peaks for Atf3, Fosb, and Fosl2. For all three of these AP-1 subunits, enrichment was much higher after 48 hours of TGFβ1 treatment, confirming their inducible expression and function during EMT (Fig. 4B and fig. S4, B and C). Atf3 and Fosb had reduced enrichment in sg*Pbrm1* cells; in contrast, Fosl2 enrichment was increased (Fig. 4B and fig. S4, B and C) consistent with data from melanoma cells with loss of PBAF (*25*). To identify whether any of the AP-1 subunits directly cooperate with PBAF, we performed overlap analysis on the genomic regions identified for each protein. Only Atf3 peaks had high overlap and coenrichment with Phf10 peaks (Fig. 4C and fig. S4, D and E). Supporting this coenrichment, Atf3 peaks were preferentially enriched at promoters, like Phf10 peaks, while Fosb and Fosl2 peaks were more enriched at intronic and intergenic regions (fig. S4F). Many of the inducible Phf10 sites, such as the promoter of *Tnfsf13b* (Fig. 3L), have similar profiles for Phf10 and Atf3 binding (Fig. 4D), supporting Atf3’s dependency on PBAF for chromatin binding.

To further understand the functional relationship between Atf3 and PBAF, we performed RNA-seq on NMuMG cells depleted for *Atf3* under normal conditions and following 48 hours of TGFβ1 treatment (fig. S4, G and H). There was a significant correlation between the DEGs in sh*Atf3* and sg*Pbrm1* cells treated with TGFβ1 (Fig. 4E), with many overlapping DEGs (fig. S4I). Pathway analysis on genes increased by sh*Atf3* and sg*Pbrm1* under TGFβ1 treatment enriched for negative regulation of cell migration and epithelial tube morphogenesis, while the genes decreased by sh*Atf3* and sg*Pbrm1* enriched for processes related to cell cycle (Fig. 4E). To determine whether Atf3 is also required in NMuMG cells during EMT, we assessed cell viability upon TGFβ1 treatment. sh*Atf3* cells exhibited lower cell survival relative to control, like cells with *Pbrm1* depletion (Fig. 4F). These results were consistent between two separate hairpins against Atf3 (fig. S4J). Overall, these results reflect a role for Atf3 in proliferation and viability during EMT.

The data above strongly suggest that Atf3 is dependent on Pbrm1 for chromatin binding (Fig. 4, B to D. However, Atf3 is a stress-inducible TF up-regulated by TGFβ1 treatment (*31*, *45*, *46*), and its induction was reduced by *Pbrm1* deletion (fig. S4A). Like *Atf3* mRNA expression levels, TGFβ1-induced protein levels of Atf3 were also blunted upon deletion of *Pbrm1* (fig. S4K). Therefore, although Atf3 cobinds with PBAF across the genome, the reduction in Atf3 binding upon *Pbrm1* deletion could be due to decreased Atf3 protein levels. To evaluate whether the decrease in Atf3 expression in sg*Pbrm1* cells is solely responsible for the observed phenotypes, we overexpressed human ATF3 in sgCt and sg*Pbrm1* cells (fig. S4L). Atf3 overexpression did not rescue the cell viability defect in sg*Pbrm1* cells upon TGFβ1 treatment, suggesting that PBAF plays a role in both Atf3 regulation as well as function in NMuMG cells (fig. S4M).

Atf3, like other AP-1 subunits, cooperates with additional TFs to regulate gene expression (*43*). To identify potential cell type–specific TFs that cooperate with Atf3 and PBAF to regulate TGFβ1-dependent genes in NMuMG cells, we again turned to the DiffTF data to identify consensus TF sites with reduced accessibility specifically in TGFβ1-treated sg*Pbrm1* cells. Unlike AP-1 sites that have decreased (Jun) or increased (Fos) accessibility in both untreated and treated sg*Pbrm1* cells, consensus TF sites associated with Irf and Snail TFs were decreased in sg*Pbrm1* cells only when treated with TGFβ1 (Fig. 4G). The interferon regulatory factor (IRF) family of TFs are established mediators of interferon (IFN) signaling pathways (*47*), and the Snail family primarily mediates signals originating from TGFβ1 stimulation (*48*). To identify which specific members of these two families are involved in EMT in NMuMG cells, we again turned to the RNA-seq datasets. Of the nine Irf family members, only Irf1 is induced by TGFβ1 in NMuMG cells (fig. S4N). In NMuMG cells, knockdown of *Irf1* increased proliferation in untreated cells but decreased viability under TGFβ1 treatment (*49*), consistent with our observations in *Pbrm1*-depleted cells. Using published ChIP-seq data of Irf1 in NMuMG cells with 48 hours of TGFβ1 treatment (*49*), we identified 2640 Irf1 peaks, of which ∼30% were cobound by Phf10 (Fig. 4, H and I) including Pbrm1-dependent inducible genes such as *Il15* (Fig. 4J), *Cxcl10*, *Jak2*, and *Csf1.*

We similarly used the RNA-seq datasets to define the expression levels of the three Snail TF family members in NMuMG cells during EMT. Only *Snai1* was induced with TGFβ1 in NMuMG cells (fig. S4O). Using published ChIP-seq data of *Snai1* from mouse mesenchymal pBI.3G breast cancer cells (*50*), we identified 28,375 *Snai1* peaks, of which ∼55% were cobound by Phf10 in TGFβ1-treated NMuMG cells (Fig. 4, K and L), including many sites with inducible Phf10 recruitment. One of these sites is Snai2, which is repressed by Snai1 at later stages of EMT (Fig. 4M) (*51*). In agreement with the strong overlap between these TFs and Phf10, both Irf1 and Snai1 peaks are also enriched for promoters (fig. S4P). Collectively, these data provide evidence that PBAF facilitates the binding of TGFβ1-inducible TFs involved in proliferation, survival, migration, and immunosuppression during EMT.

### Pbrm1 bromodomains recognize H3K14ac

**Fig. 5.**
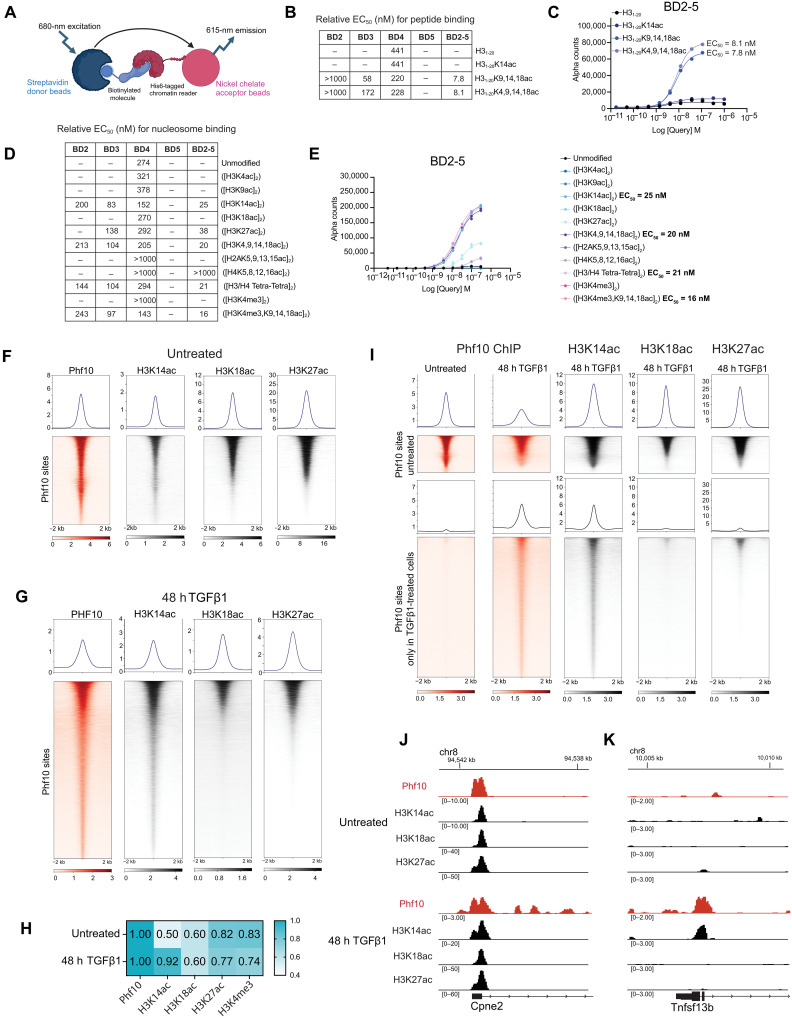
Pbrm1 binds H3K14ac at TGFβ1-inducible PBAF binding sites. (**A**) Schematic representation of EpiCypher’s Captify assay. Created in BioRender. Dykhuizen, E. (2026) https://BioRender.com/gyawlro. (**B**) Table of EC_50_ values (in nanomolars) of the different BDs for the indicated peptides obtained using the Captify assay. (**C**) Binding curves of tandem BD2-5 with the indicated peptides obtained using the Captify assay. EC_50_ (in nanomolars) values are indicated next to the corresponding curves. (**D**) Table of the relative EC_50_ values (in nanomolars) of the different BDs for nucleosomes bearing the indicated histone modifications obtained using the Captify assay. (**E**) Binding curves of tandem BD2-5 for nucleosomes bearing the indicated peptides obtained using the Captify assay. EC_50_ values (in nanomolars) obtained for positive binders are indicated in the legend. (**F** and **G**) Metagene plots and heatmaps of Phf10, H3K14ac, H3K18ac, and H3K27ac enrichment at Phf10 binding sites in untreated (F) and 48-hour TGFβ1-treated (G) NMuMG sgCt cells. (**H**) Correlation matrix with *r* values between Phf10 and H3K14ac, H3K18ac, H3K27ac, and H3K4me3 ChIP-seq enrichment in untreated and 48-hour TGFβ1-treated NMuMG sgCt cells. (**I**) Metagene plots and heatmaps of Phf10, H3K14ac, H3K18ac, and H3K27ac enrichment in untreated and 48-hour TGFβ1-treated NMuMG sgCt cells. Top: Phf10 binding sites in untreated cells. Bottom: Phf10 binding sites from TGFβ1-treated, but not untreated, cells. (**J** and **K**) Genomic tracks of Phf10, H3K14ac, H3K18ac, and H3K27ac enrichment in untreated (J) and 48-hour TGFβ1-treated (K) NMuMG sgCt cells at constitutive locus *Cpne2* and an inducible locus *Tnfsf13b*.

We next sought to define how PBAF is targeted to specific sites during EMT. We previously demonstrated that only singly modified H3_1–30_K14ac and K14ac-containing multiply acetylated peptides (H3_1–30_K14acK18acK23acK27ac) were capable of enriching the PBAF complex from nuclear lysates (*40*). To evaluate Pbrm1’s contribution to this histone mark recognition, we expressed recombinant BDs 2, 3, 4, and 5 and tandem BD2 to BD5 (BD2-5) for in vitro analysis (fig. S5A). Previous studies indicate that BD2 through BD5 are the critical bromodomains for Pbrm1 interactions in cells (*40*) and in vitro (*52*). Using Captify (Fig. 5A), peptide binding assays revealed that none of the BDs bound tightly to H3_1–20_K14ac alone; however, all queries except BD5 bound to peptides containing multiple acetylations (Fig. 5B and fig. S5B), with the highest affinity interaction between tandem BD2-5 and H3_1–20_K4acK9acK14acK18ac peptides [relative median effective concentration (EC_50_^rel^): ∼8 nM] (Fig. 5C). Although these peptide experiments indicate that PBAF/Pbrm1 may recognize multiple acetylations on H3, histone postone translational modifications (PTM) recognition can differ substantially in the nucleosome context (*53*), particularly in light of our recent finding that several Pbrm1 BDs bind nucleic acids (*39*). We therefore used Captify to screen a collection of designer nucleosomes using individually optimized conditions (see Materials and Methods). BD2 showed specificity for nucleosomes with K14ac, while the other BDs recognized multiple acetylation marks (fig. S5C) (*54*). EC_50_^rel^ values were calculated for 11 acetylated nucleosomes and an unmodified control (Fig. 5D and fig. S5D). As expected, the lowest EC_50_^rel^ values (∼20 nM) were observed for the tandem BD2-5 (Fig. 5D). Consistent with peptide pulldowns, only nucleosomes with H3K14ac were enriched. Unlike peptide experiments, however, there was very little increase in binding affinity for nucleosomes with multiple acetylation marks compared to H3K14ac alone (Fig. 5E). This indicates that, in the context of the nucleosome, Pbrm1 primarily recognizes H3K14ac via BD2 with the other bromodomains contributing affinity through H3K14ac and nucleic acids but minimally with other acetylation marks.

### Pbrm1 facilitates PBAF recruitment to sites of TGFβ1-induced H3K14ac

To elucidate the histone marks recognized by PBAF in a cellular context, we performed ChIP-seq in NMuMG cells for H3K14ac, H3K18ac, and H3K27ac. Consistent with PBAF binding at active promoters, all three histone marks were similarly enriched at Phf10 binding sites in untreated cells (Fig. 5F); however, in TGFβ1-treated cells, H3K14ac was more highly enriched at Phf10 binding sites (Fig. 5G). This was confirmed by calculating correlation scores between ChIP-seq datasets for histone marks and Phf10. While all three histone acetylation marks, as well as H3K4me3, correlate with Phf10 binding in untreated cells to a similar degree, H3K14ac is more highly correlated than other acetylation marks to Phf10 in TGFβ1-treated cells (Fig. 5H). Under TGFβ1 treatment, all three acetylation marks are maintained at constitutive Phf10 sites, while only H3K14ac is present at inducible Phf10 sites (Fig. 5, I to K). These findings strongly suggest that Pbrm1 is required for recruiting PBAF to the H3K14ac histone marks at TGFβ1-inducible sites, where it is present in the absence of other H3 acetylation marks.

### PBRM1 in human breast cancer metastasis

**Fig. 6.**
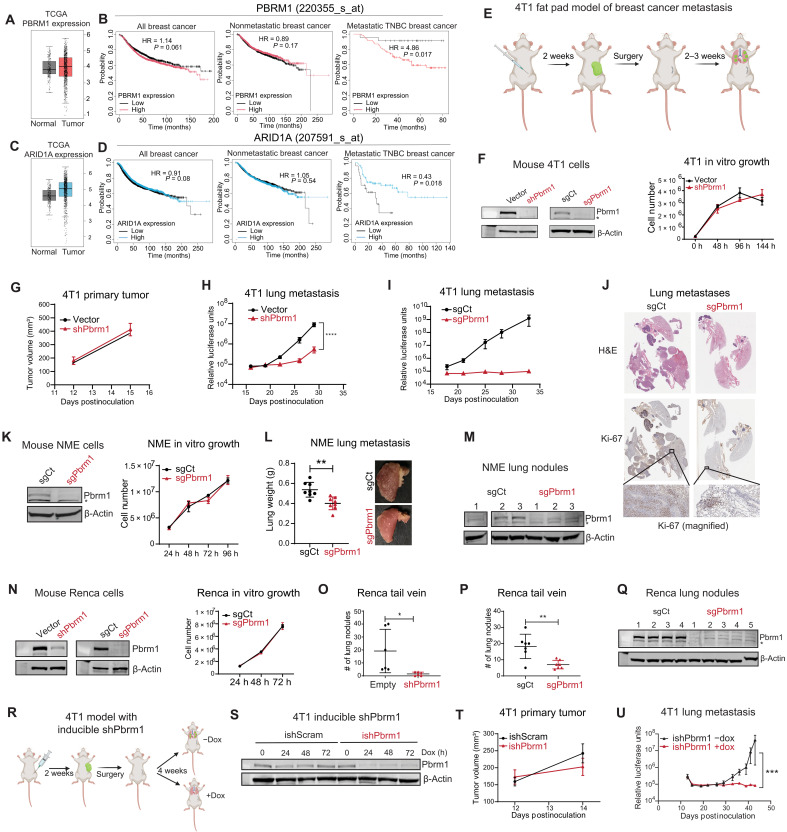
Pbrm1 is required for breast cancer metastasis in mice. (**A** and **C**) Box plots of *PBRM1* (A) and *ARID1A* (C) expression from the TCGA human breast cancer dataset, generated using GEPIA. HR, hazard ratio. (**B** and **D**) Kaplan-Meier curves of patients with breast cancer stratified by *PBRM1* (B) or *ARID1A* (D) expression, for all breast cancers (left), nonmetastatic breast cancer (middle), and metastatic TNBC (right). Curves generated using Kaplan-Meier plotter. (**E**) Schematic of the syngeneic 4T1 orthotopic fat pad model of breast cancer metastasis. Created in BioRender. Dykhuizen, E. (2026) https://BioRender.com/4az8lnc. (**F**) Left: Immunoblots of 4T1 lysates. Right: In vitro proliferation of 4T1 cells (*n* = 2 biological replicates; mean ± SD; two-way ANOVA). (**G**) Primary 4T1 tumor growth measured by calipers (mean ± SEM; two-way ANOVA). (**H** and **I**) Bioluminescent imaging following primary tumor removal in mice bearing sh*Pbrm1* or control (H) and sg*Pbrm1* or sgCt 4T1 cells (I) (mean ± SEM; two-way ANOVA). (**J**) Representative hematoxylin and eosin (H&E) and Ki-67 immunohistochemistry of lung sections from (I). (**K**) Left: Immunoblot of NME lysates. Right: NME cell counts. *n* = 2 biological replicates; mean ± SD; two-way ANOVA). (**L** and **M**) Lung weights after NME metastasis (mean ± SD; Welch’s *t* test), representative images (L), and immunoblots of ex vivo–cultured lung metastases (M). (**N**) Left: Immunoblot of Renca lysates. Right: Renca cell counts (*n* = 3 biological replicates; mean ± SD; two-way ANOVA). (**O** to **Q**) Lung nodule counts (mean ± SD; Welch’s *t* test) after Renca tail vein injections (O and P). Immunoblots of ex vivo–cultured lung metastases (Q). (**R** to **U**) Schematic of inducible sh*Pbrm1* in 4T1 metastases (R), immunoblot of 4T1 after doxycycline (Dox; 2 μg/ml) (S), primary tumor growth using calipers (T), and pulmonary bioluminescent imaging (U). (*n* = 7 or 5 biological replicates; mean ± SEM; two-way ANOVA). *P < 0.05, **P < 0.01, ***P < 0.001, and ****P < 0.0001.

Given our findings about Pbrm1 during EMT, we sought to determine whether Pbrm1 is required for metastasis. Pbrm1 was previously implicated as a tumor suppressor in breast cancer (*55*), with a low rate of mutation in patients (1.2%) (*21*) and a decrease in protein levels in cancer tissue compared to normal breast tissue in a small cohort of 150 patients (*56*). Using the larger The Cancer Genome Atlas (TCGA) breast cancer dataset, we found that *PBRM1* mRNA expression is slightly higher in breast cancer tissues compared to normal counterparts (Fig. 6A). *PBRM1* expression levels were not associated with disease progression in all breast cancers or early-stage breast cancer (Fig. 6B, left, middle). In contrast, in metastatic triple-negative breast cancer (mTNBC), *PBRM1* expression is highly predictive of worse overall survival (Fig. 6B, right). These findings suggest a context-dependent requirement for *PBRM1* in the metastatic setting. Along these lines, we found that *PBRM1* depletion does not affect the viability of the nonmetastatic MCF10CA1h (CA1h) human breast cancer cell line but is required for viability in the isogenic and metastatic MCF10CA1a (CA1a) cell line (fig. S6, A to C). cBAF-specific subunit *ARID1A* also showed elevated expression in breast cancer tissues relative to the normal group (Fig. 6C); however, in contrast to *PBRM1* expression, higher *ARID1A* expression was associated with better survival in mTNBC (Fig. 6D). This is in agreement with ARID1A as a tumor suppressor in breast cancer, where mutations are found in ∼35% of patients with TNBC (*57*). Mechanistically, ARID1A is required for estrogen receptor (ER) gene expression, and loss of *ARID1A* in luminal ER^+^ breast cancers induces a luminal-to-basal transition to a more aggressive phenotype (*58*, *59*), suggesting that cBAF and PBAF play differing roles within subtypes of breast cancer. On the basis of these data from TCGA patient samples and human cell lines, we hypothesized that *PBRM1* expression may contribute to progression of metastatic TNBC.

### Pbrm1-depleted cells exhibit reduced metastasis in mouse models

To evaluate the role of Pbrm1 in metastasis in vivo, we used the 4T1 model of breast cancer metastasis. 4T1 cells expressing luciferase were orthotopically implanted into the mammary fat pads of syngeneic Balb/c mice. When the primary tumor reached a group average of ∼400 mm^3^ in size (∼2 weeks), it was surgically removed, and the outgrowth of lung metastases was monitored using in vivo bioluminescence imaging (Fig. 6E). *Pbrm1* depletion or deletion did not significantly affect 4T1 cell proliferation in vitro (Fig. 6F) or in the orthotopic primary tumor (Fig. 6G and fig. S6D). In contrast, *Pbrm1* depletion or deletion resulted in a significant reduction of pulmonary metastasis as measured by bioluminescence (Fig. 6, H and I), which is consistent with end-point necropsy (Fig. 6J). To determine whether this dependency is unique to PBAF, we generated 4T1 cell lines with knockdown of a second PBAF subunit, *Brd7*, or a cBAF-specific subunit, *Arid1A* (fig. S6E), and similarly evaluated primary tumor growth and metastasis in vivo. There were no significant differences observed in the primary tumor growth with sh*Brd7* or sh*Arid1a* (fig. S6, F and G). In metastatic tumor growth, there was a decrease in the sh*Brd7* group (fig. S6, H and J) but not in the sh*Arid1A* group (fig. S6, I and J), further supporting different roles for cBAF and PBAF in breast cancer subtypes. We also tested *Pbrm1* depletion in the 4T1 isogenic cell line, 4T07, which forms pulmonary tumors after tail vein injection (*60*). Consistent with our findings in the 4T1 model, depletion of *Pbrm1* in the 4T07 cells similarly reduces pulmonary tumor formation (fig. S6, K and L).

We next tested the effects of *Pbrm1* depletion in additional models of metastasis. We observed similar effects in metastasis from NME (NMuMG transformed with EGFR) (*61*) tumors in NOD-Rag1 null IL2rγ null (NRG) immunocompromised mice (Fig. 6, K and L, and fig. S6M) and found that metastases from the sg*Pbrm1* tumors had higher Pbrm1 expression relative to the parental line (Fig. 6M), suggesting that metastases were enriched for populations of cells with residual Pbrm1 expression. We also observed a similar reduction in pulmonary tumors after tail vein injection with mouse syngeneic kidney cancer cell line Renca with *Pbrm1* deletion in the in Balb/c mice (Fig. 6, N to Q, and fig. S6, N to Q) and in nu/nu mice (fig. S6R).

These findings strongly suggest that Pbrm1 facilitates metastasis in vivo but do not provide clear insight into whether Pbrm1 is required for dissemination from the primary tumor, initiation, or outgrowth of pulmonary metastases. Therefore, we developed a 4T1 cell line with inducible expression of sh*Pbrm1* upon doxycycline administration (Fig. 6, R and S). These inducible sh*Pbrm1* cells were implanted into the mammary fat pads of Balb/c mice, and primary tumors were allowed to grow for 2 weeks before surgical removal (Fig. 6T). After cellular dissemination and removal of the primary tumor, mice were administered doxycycline, which does not affect pulmonary tumor growth (fig. S6S). Consistent with our constitutive depletion models, doxycycline-induced depletion of Pbrm1 after primary tumor removal also reduced metastases (Fig. 6U), indicating that Pbrm1 is required for the outgrowth of cells at the metastatic site.

### Pbrm1-mediated function in TNBC cells

**Fig. 7.**
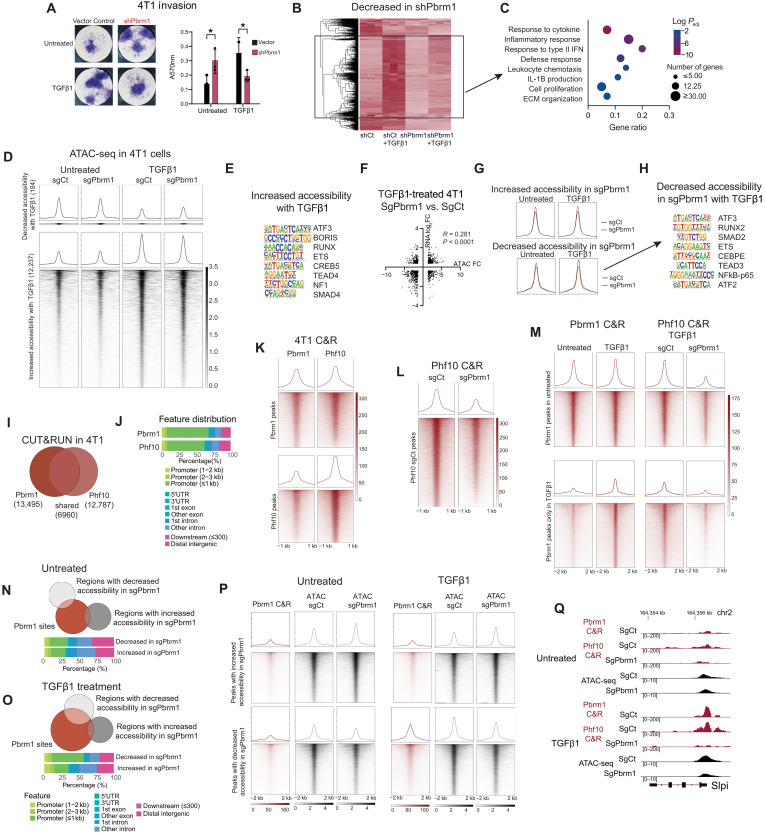
Pbrm1 is required for TGFβ1-inducible gene expression in 4T1 cells. (**A**) Representative images and quantification of Transwell invasion assays for 4T1 cells (*n* = 3 technical replicates; mean ± SD). (**B**) Heatmap of genes increased or decreased in sh*Pbrm1* 4T1 cells relative to control cells under untreated and 96-hour TGFβ1 treatment. (**C**) Gene Ontology terms enriched among the highlighted gene subsets from (B). (**D**) Metagene plots of regions with decreased (top) or increased (bottom) accessibility in TGFβ1-treated versus untreated sgCt cells. (**E**) Motif enrichment analysis [HOMER (Hypergeometric Optimization of Motif EnRichment)] of regions with increased accessibility in sgCt cells upon TGFβ1 treatment. (**F**) Scatterplot comparing ATAC-seq fold change with nearest gene RNA-seq log_2_FC for sites of differential accessibility in sg*Pbrm1* versus sgCt cells treated with TGFβ1. (**G**) Metagene plots of regions with increased (top) or decreased (bottom) accessibility in sg*Pbrm1* versus sgCt cells across all treatment conditions. Red lines denote sg*Pbrm1*, and black lines denote the sgCt signal. (**H**) Motif analysis (HOMER) of regions with reduced accessibility in sg*Pbrm1* versus sgCt cells with TGFβ1. (**I** and **J**) Overlap and genomic feature distribution of Pbrm1 and Phf10 CUT&RUN peaks in sgCt cells. (**K**) Metagene plots and heatmaps of Pbrm1 (top) and Phf10 (bottom) CUT&RUN enrichment at their respective binding sites from (I). (**L**) Metagene plots and heatmaps of Phf10 CUT&RUN enrichment in sgCt and sg*Pbrm1* cells at Phf10 ChIP-seq sites from (I). (**M**) CUT&RUN enrichment of Pbrm1 (left) and Phf10 (right) plotted at constitutive and TGFβ1-induced Pbrm1 sites. (**N** and **O**) Overlap between Pbrm1 CUT&RUN peaks and regions of altered accessibility in sg*Pbrm1* versus sgCt cells under untreated (N) or TGFβ1-treated (O) conditions, with genomic feature distributions. (**P**) Metagene plots and heatmaps of Pbrm1 CUT&RUN (C&R) and ATAC-seq signal at sites of differential accessibility in sg*Pbrm1* cells under untreated and TGFβ1-treated conditions. (**Q**) Genome tracks at the *Slpi* locus.

To evaluate how the mechanistic insights for Pbrm1 in NMuMG cells undergoing EMT relate to the requirement for Pbrm1 in 4T1 metastasis, we performed mechanistic data in 4T1 cells in vitro. Although mouse TNBC 4T1 cells are already metastatic, they have a dynamic epithelial/mesenchymal phenotype that contributes to its aggressive nature and the ability to respond to TGFβ1. In agreement with the findings in NMuMG cells, sh*Pbrm1* in untreated 4T1 cells increased invasive capabilities, while sh*Pbrm1* reduced invasive capabilities in TGFβ1-treated cells (Fig. 7A). We performed RNA-seq on 4T1 cells treated with TGFβ1 (5 ng/ml) for 96 hours and identified 500 to 1000 genes differentially regulated in sh*Pbrm1* cells compared to vector control (fig. S7, A and B). Like NMuMG cells, a subset of TGFβ1-inducible genes fails to be induced in the absence of *Pbrm1* (Fig. 7B) and are involved in processes such as cell proliferation, response to cytokines, migration, and extracellular matrix (Fig. 7C). We confirmed these findings in 4T1 cells with *Pbrm1* knockout, where we observed similar gene changes with TGFβ1 (fig. S7C), similar gene changes with sg*Pbrm1* and sh*Pbrm1* (fig. S7D), and a similar failure to induce TGFβ1-dependent genes involved in inflammation and EMT (fig. S7, E and F). Using 4T1 cells with sg*Pbrm1*, we next performed ATAC-seq with and without 96 hours of TGFβ1 treatment. TGFβ1 treatment induced large changes in DNA accessibility, but unlike in the NMuMG cells, there were very few sites with decreased accessibility, only sites with increased accessibility (Fig. 7D). There was very little difference in global accessibility in cells with sg*Pbrm1* (fig. S7G); however, there was a reduction in accessibility at inducible sites (Fig. 7D). The TF consensus sequences at the sites with TGFβ1 induced accessibility were largely conserved between NMuMG and 4T1 cells, with Atf3 as the most enriched (Fig. 7E). We identified between 7000 and 11,000 sites of differential accessibility in sg*Pbrm1* cells compared with sgCt control cells and an overall correlation between changes in accessibility and expression of the nearest gene (Fig. 7F). Like NMuMG, the increased accessibility in sg*Pbrm1* cells across conditions was consistent, while the decreased accessibility was more pronounced in TGFβ-treated cells (Fig. 7G). These sites were enriched with similar TF motifs as the TGFβ1-increased sites (Fig. 7H), consistent with a role for Pbrm1 in facilitating accessibility at TGFβ1-dependent genes. To evaluate whether Pbrm1 facilitates this using a similar mechanism in 4T1 cells as NMuMG, we next performed CUT&RUN for both Pbrm1 and Phf10. As expected, Pbrm1 and Phf10 peaks have high overlap (Fig. 7I) and are enriched at promoters (Fig. 7J). Both subunits are highly enriched at their respective binding sites (Fig. 7K), which is consistent across two biological replicates (fig. S7, H and I). Phf10 enrichment is reduced in sg*Pbrm1* cells (Fig. 7L and fig. S7J), and similar to NMuMG cells, TGFβ1 treatment increases PBAF binding at inducible sites, which is abrogated upon *Pbrm1* knockout (Fig. 7M and fig. S7K). By integrating the ATAC-seq and CUT&RUN data, we find the largest overlap between PBAF binding and sites with decreased accessibility in sg*Pbrm1* cells treated with TGFβ1 (Fig. 7, N and O), a higher percentage of promoters in sites with decreased accessibility in sg*Pbrm1* cells treated with TGFβ1 (Fig. 7, N and O), and the greatest enrichment of Pbrm1 binding at sites with decreased accessibility in sg*Pbrm1* cells treated with TGFβ1 (Fig. 7P). Therefore, the mechanism for Pbrm1-dependent induction of mesenchymal genes in NMuMG cells is consistent with the mechanism for Pbrm1-dependent induction of metastasis genes in 4T1 cells, such as the TGFβ1-inducible gene *Slpi* (Fig. 7Q), which is important for TNBC metastasis (*62*). Together, Pbrm1 is required for TGFβ1-dependent gene expression in both normal murine mammary cells and TNBC cells.

## DISCUSSION

The mammalian PBAF subcomplex was biochemically defined at the same time as cBAF (*19*), yet its biochemical role in gene transcription is much less clear. Here, we find that in epithelial cells, Pbrm1 deletion slightly increases the growth rate but blocks the completion of EMT upon stimulation with TGFβ1. This requirement for EMT is consistent with the phenotype described for the Pbrm1 knockout mouse, which is embryonic lethal at embryonic day 14.5 (*63*) due to a failure in EMT during heart development (*64*, *65*). It is also consistent with reports of a requirement for Pbrm1 (*66*) and ARID2 (*67*) in BMP/TGFβ-dependent mesenchymal stem cell osteolineage differentiation and a requirement for Brd7 in the expression of TGFβ response genes (*68*). Identifying this dependency on PBAF for EMT has allowed us the opportunity to dissect the role of PBAF in facilitating gene expression. We identified more than 1000 DEGs in epithelial cells with Pbrm1 knockout; however, the ChIP-seq and ATAC-seq provided very little insight into how PBAF might be involved in the regulation of these genes. As has been observed in other cell types (*5*, *25*), PBAF is bound at promoters, with Phf10 binding correlating strongly with H3K4me3 enrichment. In NMuMG cells, we observed Phf10 binding at more than 9000 promoters; however, only about half of the DEGs have Phf10 enrichment at their promoters. Further, there was no difference between Phf10 enrichment at up-regulated compared to down-regulated genes and no correlation between Phf10 enrichment and the degree to which gene expression changes upon Pbrm1 knockout. Upon treatment with TGFβ1, however, Phf10 is recruited to new sites, where we could detect Pbrm1-dependent increases in DNA accessibility using ATAC-seq. Further, binding studies with recombinant nucleosomes reveal that, contrary to the results with histone peptides, only H3K14ac is important for Pbrm1 binding, which was supported by Phf10 enrichment at inducible sites marked only by H3K14ac. Although acetylation marks on histone 3 tails are often deposited indiscriminately by Gcn5 (*69*) and found together at promoters, H3K14ac is found alone at a subset of inducible genes (*70*, *71*), indicating that PBAF activity may be more important for the initiation, rather than maintenance, of promoter accessibility. Uncovering the specific acetyltransferase responsible for depositing H3K14ac and how its activated upon TGFβ1 treatment will be useful for further establishing a role for PBAF in gene induction.

EMT is an important part of cancer metastasis, and we find that Pbrm1 dependency extends to the metastasis of 4T1 mouse breast cancer cells from the primary site to the lung. Using inducible *Pbrm1* knockdown, we establish that Pbrm1 is important for outgrowth of lung metastases after dissemination. These findings are consistent with our previous work demonstrating that cells must undergo an EMT before initiation of metastatic tumor growth (*72*). What remains to be seen is whether deleting Pbrm1 in established metastatic tumors could halt or even regress metastatic tumor growth. In vitro, deletion of *Pbrm1* prevents the induction of a subset of TGFβ1 response genes in metastatic TNBC cells using a similar mechanism as defined in NMuMG cells. In particular, the loss of Pbrm1 has a very pronounced effect on the expression of immunosuppressive (*73*) IFN-γ response genes during EMT. Pbrm1 regulation of IFN-γ response genes was similarly observed in human metastatic TNBC cells (*74*) and metastatic prostate cancer cells (*75*). This may implicate some contribution of the immune system in the reduction of metastases in *Pbrm1*-depleted tumors. Consistent with this, PBAF subunit deletions in tumors increase T cell killing (*76*) and predict a better response to immunotherapies (*77*, *78*). This was originally hypothesized to be the result of an increase in IFN response in Pbrm1-mutant cells, through Pbrm1-mediated gene repression (*79*), Pbrm1-mediated DNA damage repair (*80*), or Pbrm1-mediated alleviation of replication stress (*81*, *82*); however, the expression of immunosuppressive genes may also contribute. Paradoxically, the loss of PBAF subunits can also result in immunologically cold tumors in some cases, which is also attributed to the expression of IFN-γ response genes (*83*). Therefore, the context-dependent roles for Pbrm1 in cancer progression may reflect, in part, the context-dependent roles for IFN-γ in cancer progression (*84*, *85*). Additional studies will be required to establish how Pbrm1 facilitates IFN-γ response in tumors to facilitate metastasis and immune suppression and the context in which pharmacologically targeting PBAF could be therapeutically advantageous.

### Limitations of this study

TGFβ1 in NMuMGs induces EMT if cells are in G_1_/S and induces apoptosis if cells are in G_2_/M (*29*). Some of the phenotypes, and possibly gene expression changes, we observe with NMuMG cells may be related to the slightly increased proliferation rate or increased DNA damage (*86*) in the Pbrm1 knockout that increases apoptosis rates. Nevertheless, even the populations in G_1_/S fail to fully transition to a mesenchymal state and resume proliferation. This could be due to a role for PBAF in activating genes important for replication during G_1_, as has been observed for cBAF in SWI/SNF-dependent cancers (*87*), or could be due to the induction of cell cycle checkpoints due to a failure in stress response (*22*), DNA damage repair (*26*, *80*), or mitosis (*80*, *88*, *89*). The use of an inducible recruitment (*7*) or inducible degron system would help separate direct from indirect gene targets, as well as transcriptional roles in G_1_ versus function during G_2_/M.

While we observe a strong dependency on Pbrm1 for TNBC metastasis, this is likely context dependent. In renal cancers, *PBRM1* is mutated as an early event in the primary tumor after *Von Hippel Lindau* (*VHL*) mutation (*90*). In addition, while *PBRM1*-mutant ccRCC tumors are much less metastatic (*91*), they can eventually metastasize. In addition, recent reports find that Brd7 deletion in the 4T07 dormant breast cancer model permits the outgrowth of metastases (*92*), implying that PBAF is not required for all metastatic tumors. Nevertheless, many reports indicate that Pbrm1 mutant tumors respond better to a variety of therapies (*78*, *81*, *93*), implicating that targeting Pbrm1 as part of a combination therapy may be an effective treatment for metastatic disease. Further studies with pharmacological inhibitors of PBAF would resolve whether it is a potential therapeutic target in metastatic cancers as well as possibly in T cells to decrease exhaustion (*94*, *95*).

## MATERIALS AND METHODS

### Cell lines and culture conditions

NMuMG cells were purchased from American Type Culture Collection and grown in Dulbecco’s modified Eagle’s medium (DMEM; Corning) supplemented with 10% fetal bovine serum (Corning), insulin (10 μg/ml; Sigma-Aldrich), 1% l-glutamine (Corning), 1% antibiotics [penicillin (100 U/ml) and streptomycin (100 g/ml); Corning], and 1% sodium pyruvate (Corning).

The epidermal growth factor receptor transformed NME cell line with stable luciferase expression has been reported before (*61*) and was cultured similar to NMuMG cells as described above. 4T1 and 4T07 cells stably expressing luciferase (*60*, *96*) were grown in DMEM (Corning) supplemented with 10% fetal bovine serum (Corning), 1% l-glutamine (Corning), 1% antibiotics [penicillin (100 units/ml) and streptomycin (100 g/ml); Corning], and 1% sodium pyruvate (Corning). Renca cells were grown in RPMI 1640 (Corning) supplemented with 10% fetal bovine serum (Corning), 1% non-essential amino acids (Corning), 1% l-glutamine (Corning), 1% antibiotics [penicillin (100 units/ml) and streptomycin (100 g/ml); Corning], and 1% sodium pyruvate (Corning). MCF10A-CA1h, MCF10A-CA1a, and human embryonic kidney (HEK) 293T were cultured in DMEM (Corning) supplemented with 10% fetal bovine serum (Corning), 1% l -glutamine (Corning), 1% antibiotics [penicillin (100 units/ml) and streptomycin (100 g/ml); Corning], and 1% sodium pyruvate (Corning).

All cell lines were grown at 37°C in a humidified atmosphere in a 5% CO_2_ incubator. All the media were supplemented with 1:10,000 dilution of Plasmocin (InvivoGen) and routinely checked for mycoplasma contamination.

### Generation of cell lines

Constitutive knockdown was performed using shRNA-mediated knockdown with lentiviral construct pLKO.1. Doxycycline-inducible knockdown was performed using the lentiviral construct pLKO-tet-ON (*97*). The shRNA constructs contain the following mature antisense sequences: mouse Pbrm1: (TRCN0000081820) TTCTAGGTTGTATGCCTGTCG; mouse Brd7: (TRCN0000030015) ATAATCATGGAGTAGCCAGGC; mouse Arid1A: (TRCN0000071395) ATTGTAGGTCATGTCATTTCG; mouse Atf3: (TRCN0000082131) TTGTTTCGACACTTGGCAGCA; scramble control hairpin: GCTACACTATCGAGCAATT.

NMuMG *Pbrm1* knockout (KO) cell line generation has been described before (*22*). The same constructs and methodology were used for generating Renca, 4T1, and NME *Pbrm1* KO cell lines.

Renca parental cells were first transduced with lentiviral particles for the dual reporter construct pFU-Luc2-eGFP (L2G) (*98*) (a gift from H. Lui), and the green fluorescent protein (GFP)–expressing cells were selected using fluorescence-activated cell sorting. These GFP-positive cells were then transduced with either shRNA lentiviral particles or used for generating Pbrm1 KO cells. Atf3 reexpression was done by transient transfection using the Addgene construct #26115 (pRK-ATF3 was a gift from Y. Ye) (*99*), and stable clones were selected by continuous G418 administration (600 μg/ml) and used for experiments.

After performing genetic perturbation, cell line growth rates were compared by seeding a fixed number of cells on day 0 and then counting cells using trypan blue at the indicated time points. For longer time frames, a proportion of cells was reseeded at the time of counting for subsequent time points. Two-way analysis of variance (ANOVA) was done for statistical comparison.

### Lentiviral infection

HEK293T cells were transfected with knockdown lentivirus constructs along with packaging vectors (pMD2.G and psPAX2 for pLKO.1; pCMV-8.2∆VPR and pCMV-VSV-G for pLKO-tet-ON). After 48 hours, the supernatant was collected and concentrated by ultracentrifugation [17,500 rpm with Beckman rotor SW 32 Ti/∼52,000*g* and resuspended in 200 μl of phosphate-buffered saline (PBS)]. Cells were transduced with concentrated virus using spinfection (1500 rpm/∼250*g* in swing bucket centrifuge for 1 hour) and incubated for 48 hours. Cells were selected with puromycin (2 μg/ml) (Sigma-Aldrich) and hygromycin (200 μg/ml) (Corning) where applicable. The efficiency of all constructs was confirmed by immunoblotting.

### Immunoblotting

Cells were given the requisite treatments for the indicated time periods, followed by harvesting by trypsinization. Whole-cell extracts were prepared by resuspending cell pellets in radioimmunoprecipitation assay buffer (RIPA) [50 mM tris (pH 8.0), 150 mM NaCl, 0.1% SDS, 0.5% Na deoxycholate, and 1% NP-40], supplemented with freshly added phenylmethylsulfonyl fluoride (PMSF), aprotinin, leupeptin, and pepstatin, and incubated for 30 min at 4°C. Nuclear lysates were prepared by resuspending cells in buffer A [20 mM Hepes (pH 7.9), 25 mM KCl, 10% glycerol, 0.1% NP-40 with PMSF, aprotinin, leupeptin, and pepstatin] at a concentration of 20 million cells/ml. The cells were kept on ice for 5 min, and nuclei were isolated by centrifugation at 600*g* for 10 min. The nuclei pellet was resuspended in chromatin IP buffer [20 mM Hepes (pH 7.9), 150 mM NaCl, 1% Triton X-100, 7.5 mM MgCl_2_, 0.1 mM CaCl_2_ with PMSF, aprotinin, leupeptin, and pepstatin]. The lysates were centrifuged at 21,000*g* for 30 min at 4°C, and the supernatants were preserved. Protein concentration estimations for the supernatants were performed using the bicinchoninic acid (BCA) protein assay kit (Pierce Biotechnology) with bovine serum albumin (BSA) as the standard. Lysates containing equal amounts of protein were mixed with 4× lithium dodecyl sulfate sample buffer containing 10% 2-merchaptoethanol. The proteins were denatured for 5 min at 95°C, separated on a 4 to 12% bis-tris gradient protein gel, and transferred to polyvinylidene difluoride membrane. The membrane was blocked with 5% BSA in tris-buffered saline containing 0.1% Tween 20 for 30 min at room temperature (RT) and then incubated with primary antibodies overnight at 4°C. The primary antibodies were detected by incubating the membranes in goat anti-rabbit or goat anti-mouse secondary antibodies (LI-COR Biotechnology, Lincoln, NE) conjugated to IRDye 800CW or IRDye 680RD, respectively, for 1 hour at RT, and the signals were visualized using an Odyssey Clx imager (LI-COR Biotechnology).

### Immunoprecipitation

Cell treatments and nuclear lysates were prepared in chromatin IP buffer as described above and treated with TURBO deoxyribonuclease (4 U/ml; Ambion) for 30 min at 4°C. The extracts were cleared by centrifugation 21,000*g* at 4°C for 30 min. Protein quantification was performed on the clarified supernatants using a BCA protein assay kit (Pierce Biotechnology) with BSA as the standard. For ATF3 IP, 5% input was taken out, and 500 μg of nuclear lysate per sample was used for setting up IP using ATF3 antibody (ab207434, Abcam) or normal immunoglobulin G (IgG; 2729S, Cell Signaling Technology) and rotated overnight at 4°C. Equilibrated protein A beads (20 μl per IP) were added to the lysates and rotated at 4°C for another 2 hours. The bead-bound complexes were washed twice with chromatin IP buffer and thrice with high stringency buffer [20 mM Hepes (pH 7.9), 500 mM NaCl, 1% Triton X-100, 0.5% sodium deoxycholate, 1 mM EDTA with PMSF, aprotinin, leupeptin, and pepstatin] followed by boiling in 1× lithium dodecyl sulfate loading dye at 80°C for 10 min and immunoblotting. For PHF10 IP-MS, a 2% input was removed, and 500 μg of lysate per sample was used in an IP with PHF10 antibody (PA5-30678, Invitrogen) and rotated overnight at 4°C. Equilibrated protein A beads (15 μl per IP) were added to the lysates and rotated at 4°C for another 2 hours. The beads were washed three times with chromatin IP buffer and three times with 1× PBS.

### LC-MS sample preparation

The phase transfer surfactant buffer containing 12 mM sodium deoxycholate, 12 mM sodium lauroyl sarcosinate, 10 mM Tris(2-carboxyethyl)phosphine) (TCEP), and 40 mM chloroacetamide in 50 mM tris·HCl (pH 8.5) was added to the magnetic beads. The proteins were incubated for 10 min at 95°C and digested on beads with Lys-C (Wako) at a 1:100 (w/w) enzyme-to-protein ratio for 5 hours at 37°C. Furthermore, trypsin was added to a final 1:50 (w/w) enzyme-to-protein ratio for overnight digestion at 37°C. The supernatant containing the digested proteins was separated from the magnetic beads with the help of a magnetic rack separator and processed further. Then, a final concentration of 1% trifluoroacetic acid was added to acidify the samples. An ethyl acetate solution was added at a 1:1 ratio to the samples. The solution was vortexed for 2 min and then centrifuged at 20,000*g* for 3 min to separate the aqueous and organic phases. The top layer (the organic phases) was removed, and the aqueous phase was collected, dried down in a vacuum centrifuge, and desalted using TopTip C18 tips (Glygen) according to the manufacturer’s instructions. The desalted samples were dried completely in a vacuum centrifuge.

### LC-MS analysis

The proteomic samples were spiked with an 11-peptide Retention Time internal standard (Biognosys) to normalize the liquid chromatography–mass spectrometry (LC-MS) signal between sample runs. All samples were loaded into an Easy-nLC 1000 (Thermo Fisher Scientific) and separated with a 45-cm packed column (360-μm outer diameter × 75-μm inner diameter) containing C18 resin (2.2 μm, 100 Å; Michrom Bioresources) with a heated 30-cm column heater (Analytical Sales and Services) set to 50°C. The mobile phase buffer contained 0.1% formic acid in high-performance liquid chromatography (HPLC) grade water (buffer A) with an eluting buffer containing 0.1% formic acid in 80% (v/v) acetonitrile (buffer B) run with a linear 60-min gradient of 10 to 35% buffer B at a flow rate of 300 nl/min. The HPLC was coupled with an LTQ-Orbitrap Velos Pro mass spectrometry (Thermo Fisher Scientific). The mass spectrometry was run in data-dependent mode with a full-scan MS [from mass/charge ratio (*m/z*) 350 to 1500 with a resolution of 30,000], followed by tandem MS of the 10 most intense ions subjected to collision-induced dissociation (CID) fragmentation. CID fragmentation was performed and acquired in the linear ion trap (minimal signal threshold, 1000 counts; normalized collision energy, 30%; activation Q, 0.25; activation time, 10 ms; default charge state, 3; isolation window, 3 *m/z*; dynamic exclusion, 60 s).

### LC-MS data processing

The raw data were searched against the mouse Swiss-Prot database with no redundant entries, using Sequest and Byonic (Protein Metrics) search engines loaded into Proteome Discoverer 2.3 software (Thermo Fisher Scientific). MS1 precursor mass tolerance was set at 10 parts per million, and MS2 fragment tolerance was set at 0.6 Da. In the processing workflow, search parameters for both search engines were performed with full trypsin/P digestion, a maximum of two missed cleavages allowed, a static modification of carbamidomethylation on cysteines (+57.0214 Da), and variable modifications of oxidation (+15.9949 Da) on methionine residues and acetylation (+42.011 Da) at N terminus of proteins. The false discovery rates (FDRs) of peptide spectrum matches, peptides, and proteins were set at 0.01 (strict) and 0.05 (relaxed). All protein and peptide identifications were grouped, and any redundant entries were removed. Unique peptides and unique master proteins were reported. Last, the proteomic abundance results were further normalized using the spiked 11-peptide Retention Time internal standard.

### TGFβ1 treatment assays

NMuMG cells were seeded in six-well plates with the indicated concentrations of TGFβ1 (R&D Systems, catalog no. 7754-BH) to induce EMT and cultured for 2 to 10 days as detailed in the respective figure legends. To assess whether the EMT was reversible, the cells were first treated with TGFβ1 for 5 days, and then TGFβ1 was removed while culturing the cells in the same way as described above. NMuMG cells were trypsinized and counted using the trypan blue method every 48 to 72 hours and reseeded in six-well plates at the same cell number as day 1 at each passage. 4T1 cells were seeded in six-well plates with or without TGFβ1 (5 ng/ml) and treated for 4 days, with a passage on day 2. Cell survival was calculated as percentage of cell number relative to untreated control within the group. Each experiment was done in at least three biological replicates. Error bars represent SD, and stats were done using multiple unpaired *t* tests with Holm-Sidak correction.

### Cell cycle analysis

NMuMG cells were treated with TGFβ1 for 0 to 72 hours as described above and analyzed using the 488 EdU Click Proliferation Kit (BD Biosciences, #565455) at the indicated time points. For each time point, 10 μM EdU was added to the media 1 hour before harvesting, and the cells were processed and frozen until ready for use according to the manufacturer’s instructions. PI/ribonuclease (RNase) solution (Invitrogen, #F10797) was used for DNA staining. The cells were run on the BD Fortessa LSR flow cytometry cell analyzer, and the data were analyzed using FlowJo software. The PI intensity was used to designate G_0_/G_1_ and G_2_/M populations. Each experiment was done in three biological replicates. Error bars represent SD, and stats were done using multiple unpaired *t* tests with Holm-Sidak correction.

### Apoptosis analysis

NMuMG cells were treated with TGFβ1 for 0 to 5 days as described above and harvested using Accutase (Corning, #25-058-CI) at the indicated time points. The cells (including floating cells) were processed and stained using the FITC (fluorescein isothiocyanate) Annexin V Apoptosis Detection Kit I (BD Biosciences, #556547) according to the manufacturer’s instructions. The cells were immediately run on the BD Fortessa LSR flow cytometry cell analyzer, and the data were analyzed using FlowJo software. Each experiment was done in two biological replicates. Error bars represent SD, and stats were done using multiple unpaired *t* tests with Holm-Sidak correction. A portion of the harvested cells above was used for C-PARP [cleaved poly (ADP-ribose) polymerase] immunostaining as an additional assay for apoptosis.

### Invasion assays

NMuMG and 4T1 cells were either left untreated or treated with TGFβ1 for 5 days, before seeding on the transwells for invasion assays. The assay was done using regular Pathclear basement membrane extract (BME) or reduced growth factor Pathclear BME (R&D Systems) diluted to 10% in serum-free media, TGFβ1 in serum-free media was added to the top chamber only, 5 to 10% serum was added to the bottom chamber only, and serum-free media in both chambers was used as the baseline invasion control. The invading cells were fixed and stained with crystal violet after 48 hours of incubation. The stained cells were photographed, and the stain was redissolved in 0.2% SDS and quantitated by absorbance readings at 570 nm. The experiments were done once with three technical replicates. Error bars represent SD, and stats were done using multiple unpaired *t* tests with Holm-Sidak correction.

### In vivo studies

All animal studies were completed in compliance with Purdue University (West Lafayette, IN) animal care and use guidelines after approval by the Purdue Institutional Animal Care and Use Committee. Luciferase expressing 4T1 cells (4T1-L4) (*96*) transduced with either pLKO.1 control, *shPbrm1* (10 mice per group), *shBrd7*, *shArid1A* (5 mice per group) or sgCt, *sgPbrm1*, genetic perturbations were orthotopically engrafted onto the mammary fat pads of 4 to 6 weeks old female Balb/c mice (the Jackson Laboratory)—50,000 cells per mouse in 50 μl of 1× PBS. Primary tumor measurements were done weekly using vernier calipers, and the tumors were surgically removed 2 weeks after engraftment. Metastasis development was monitored by weekly bioluminescent imaging using the Advanced Molecular Imager (Spectral Instruments).

4T1-L4 cells expressing either doxycycline-inducible scramble or *shPbrm1* (12 mice per group) genetic perturbations were orthotopically engrafted onto the mammary fat pads of 4- to 6-week-old female Balb/c mice (the Jackson Laboratory)—50,000 cells per mouse in 50 μl of 1× PBS. Primary tumor measurements were done weekly using vernier calipers, and the tumors were surgically removed after 2 weeks of injections. Each group was then divided into no doxycycline administration (5 mice per group) or doxycycline administration in drinking water (7 mice per group) subgroups such that average primary tumor size was comparable. Doxycycline water administration (1 mg/ml + 5% sucrose in drinking water) was started 24 hours after primary tumor removal, with two changes/week until the experiment ended. Metastasis development was monitored by weekly bioluminescent imaging using the Advanced Molecular Imager (Spectral Instruments).

Control or Pbrm1-depleted (pLKO.1, *shPbrm1*; 5 mice per group) 4T07 pLuc cells (*60*) were injected into the tail veins of female Balb/c mice (1.0 × 10^6^ cells in 100 μl of 1× of PBS), and pulmonary tumor development was monitored by weekly bioluminescent imaging. Control or Pbrm1-depleted (sgCt, *sgPbrm1*) NME pLuc cells (*61*) were orthotopically engrafted onto the mammary fat pads of 15- to 16-week-old female NRG mice (Purdue Biological Evaluation core, eight mice per group)—1.1 × 10^6^ cells per mouse in 50 μl of 1× PBS. Primary tumor measurements were done weekly using vernier calipers, and the tumors were surgically removed after 4 weeks of injections. Metastasis development was monitored by weekly bioluminescent imaging. At the time of necropsy, a portion of the lungs was saved for ex vivo subculture with antibiotic selection to enrich tumor-derived cells, and immunoblotting was performed for Pbrm1 protein expression.

Control or Pbrm1-depleted (pLKO.1, *shPbrm1*; 6 mice per group) and control or Pbrm1-deleted (sgCt, *sgPbrm1*; 7 mice per group) Renca FF-GFP-pLuc cells were injected into the lateral tail vein of male Balb/c mice (100,000 cells per mouse) in 100 μl of 1× PBS, and pulmonary tumor development was monitored by weekly bioluminescent imaging. For the experiment with control and Pbrm1-deleted Renca cells, at the time of necropsy, a portion of the lungs was saved for ex vivo subculture with antibiotic selection to enrich tumor-derived cells, and immunoblotting was performed for Pbrm1 protein expression. Control or Pbrm1-depleted (pLKO.1, *shPbrm1*) Renca pLuc cells were injected into the lateral tail vein of male nu/nu mice [five mice per group (Taconic); 100,000 cells per mouse] in 100 μl of 1× PBS, and metastasis development was monitored by weekly bioluminescent imaging.

Upon necropsy, primary tumors (if applicable) and lungs from all animals were removed, fixed in 10% formalin for 24 hours, and dehydrated in 70% ethanol for visualization of pulmonary metastatic nodules and further histologic analyses. All animal studies were performed in accordance with the animal protocol procedures approved by the Institutional Animal Care and Use Committee of Purdue University. Statistical analyses were performed using Welch’s *t* test and two-way ANOVA with multiple comparisons as indicated in the figure legends

### Quantitative reverse transcription polymerase chain reaction

RNA was extracted using TRIzol (Ambion Inc.). cDNA was synthesized using a Verso cDNA synthesis kit (Thermo Fisher Scientific) using 3:1 mix of random hexamers and oligo dT primers as per the manufacturer’s recommendation. Specific targets were amplified using PowerUp SYBR Green Master Mix (Applied Biosystems) in a Bio-Rad CFX qPCR instrument. The following qPCR primers were used: mAtf3 forward primer: GAGGATTTTGCTAACCTGACACC; mAtf3 reverse primer: TTGACGGTAACTGACTCCAGC; mOaz1 forward primer: GTGGTGGCCTCTACATCGAG; mOaz1 reverse primer: GTTGCTCCTCTGCGAACTCTA.

### RNA sequencing

NMuMG cells were seeded in six-well plates with or without TGFβ1 as described above. Cells were harvested at 24, 48, and 72 hours posttreatment in TRIzol, and total RNA was isolated using the PureLink RNA isolation kit (Invitrogen, #12183018A). Downstream library construction, sequencing on NovaSeq 6000, and data analysis were done by Novogene. 4T1 cells (*shPbrm1* datasets) were seeded with or without TGFβ1 for 4 days and processed as above for the total RNA isolation. Library construction was done using the Illumina Stranded mRNA prep, ligation kit (Illumina, #20040534) according to the manufacturer’s instructions and checked for quality using Qubit and Agilent Bioanalyzer by Purdue Genomics Core Facility. Libraries were sequenced using 150-bp paired end sequencing on a NovaSeq 6000 platform (Novogene, Sacramento, CA). 4T1 cells (*sgPbrm1* datasets) were seeded with or without TGFβ1 for 4 days, and then 4 × 10^5^ cells were suspended in 100 μl of Zymo DNA/RNA Shield and sent to Plasmidosaurus for RNA-seq sample preparation. RNA isolation, library construction, and sequencing were done by Plasmidosaurus. [Briefly, mRNA is converted into cDNA via reverse transcription using a poly(dT)VN primer, followed by second-strand synthesis, and then tagmentation, library indexing, and amplification. This approach uses 3′ end counting to capture differential gene expression. The single-end sequencing is stranded and sequences toward the 3′ end. Unique molecular identifiers are used to deduplicate and unique dual indices (UDIs) to prevent index hopping. All experiments were performed in three biological replicates.

### RNA-seq analysis

NMuMG *sgPbrm1* and sh*Atf3* datasets were performed by Novogene: Raw reads were trimmed for adapter, poly-N, and low-quality sequences using fastp and mapped to mouse mm10 genome build using STAR (*100*) (v2.6.1d) software (NMuMG *sgPbrm1* datasets, mismatch = 2) or Hisat2 (*101*) (v2.0.5) software (NMuMG shATF3 datasets, default parameters). Differential expression analysis was done using DESeq2 (*102*) R package (v1.20.0), and the resulting *P* values were adjusted using the Benjamini and Hochberg’s approach for controlling the FDR. Heatmaps were visualized using Heatmap2 (*103*) and Venn diagrams were visualized using Eulerr (*104*) (v7.0.2).

For 4T1 sh*Pbrm1* datasets, raw reads were trimmed for adapter sequences and low-quality bases using trim-galore (v0.6.7) (https://doi.org/10.5281/zenodo.5127899) and mapped to mouse mm10 genome build using STAR (*100*) (v2.7.10a). Reads with MAPQ (mapping quality) >10 were used for downstream analysis. FeatureCounts from subread (v2.0.1) was used to map reads to corresponding genes according to GENECODE Mus_musculus.GRCm38.102 annotation. Differential gene expression analysis was performed by edgeR (*105*) (v3.38.0). Read counts were normalized using the trimmed mean of M-values (TMM) method, after filtering out lowly expressed genes with the filterByExpr function using its default settings. Genes with FDR-adjusted *P* values of <0.01 and absolute log_2_ fold changes of >1 were considered as DEGs.

For 4T1 sg*Pbrm1* datasets, data analysis till raw count generation was done by Plasmidosaurus. Raw reads were filtered using fastp (v0.24.0): poly-X tail trimming, 3′ quality-based tail trimming, a minimum Phred quality score of 15, and a minimum length requirement of 50 bp. Alignment was done to the mouse genome build mm39 using STAR (v2.7.11) (*100*) with noncanonical splice junction removal and output of unmapped reads. Coordinate sorting of BAM (Binary Alignment Map) files was done using SAMtools (v1.22.1) (*106*). Gene-level read counts were quantified with featureCounts subread package (v2.1.1) with strand-specific counting, multimapping read fractional assignment, exons, and three prime untranslated region as the feature identifiers and grouped by gene_id. Final gene counts were annotated with gene biotype, and other metadata were extracted from the reference GTF (Gene Transfer Format) file. Sample-sample correlations for sample-sample heatmap and principal components analysis (PCA) were calculated on normalized counts (TMM) using Pearson correlation. One replicate from each TGFβ1-treated group was excluded following PCA and pairwise sample correlation assessment, which identified these samples as outliers relative to their respective group replicates. The final dataset comprised 10 samples distributed across four conditions: untreated control (Ct_Veh, *n* = 3), TGFβ1-treated control (Ct_TGFβ1, *n* = 2), untreated sg*Pbrm1* (Sg_Veh, *n* = 3), and TGFβ1-treated sg*Pbrm1* (Sg_TGFβ1, *n* = 2). Differential expression analysis was performed using edgeR (*105*) (v3.38.4). Read counts were normalized using the TMM method after removing lowly expressed genes with the filterByExpr function at default settings. A group-level model was fit to enable testing of all pairwise contrasts between the four conditions, including the interaction term between Pbrm1 genotype and TGFβ1 treatment status. Genes with FDR-adjusted *P* values of <0.05 were considered differentially expressed. Volcano plots, heatmaps, metagene analysis plots, and summary tables were generated in R.

### ATAC-seq

NMuMG cells were seeded in six-well plates with or without TGFβ1 as described above. Cells were harvested at 24, 48, and 72 hours posttreatment and processed according to the Omni-ATAC protocol (*107*). 4T1 cells were seeded in six-well plates with or without TGFβ1 for 4 days as described above and processed according to the Omni-ATAC protocol (*107*). The final libraries were subjected to double size selection (0.5× and 1.5×) and quality checked using Qubit and Agilent Bioanalyzer/TapeStation by Purdue Genomics Core Facility. NMuMG libraries were sequenced using 150-bp PE sequencing on a NovaSeq 6000 platform (Novogene, Sacramento, CA). 4T1 libraries were sequenced using 150-bp PE sequencing on an Aviti platform (Element Biosciences) by the Purdue Genomics Core Facility. All experiments were performed with three biological replicates.

### ATAC-seq data processing

#### NMuMG datasets

Quality trimming (adapter removal, phred quality cutoff ≥ Q30, and minimum read length of 50 bp after quality filtering) of raw reads was performed using fastp (v0.19.5) tool. Trimmed reads were mapped to mouse mm10 genome build using the bowtie2 (*108*) (v2.3.3) aligner. Peak calling was performed with Genrich in ATAC-seq mode with parameters (-j -r -e chrM -m 30 -v -k -q 0.05) that account for ATAC-seq shifting, minimum MAPQ of 30, *q* value cutoff of 0.05, and removal of mitochondrial, duplicate reads, nonunique mapped reads, and improperly paired reads. Peaks were annotated using the R package ChIPseeker (*109*) (v3.15). Differential peak calling was performed using MACS2 (*110*) (v2.2.9.1) using default parameters in the galaxy interface (*103*). PCA plot was generated using DiffBind (*111*) (v3.19.0).

#### 4T1 datasets

Paired-end reads were aligned to the mouse mm10 reference genome using Bowtie2 (*108*) (v2.4.5) with ENCODE (Encyclopedia of DNA Elements) ATAC-seq standard parameters (--very-sensitive -X 2000 --no-mixed --no-discordant). Aligned reads were filtered with SAMtools (*106*) to remove unmapped, nonprimary, supplementary, and improperly paired alignments, as well as reads failing quality filters (SAMtools flags -F 1804 -f 2) and reads with mapping quality below MAPQ of 30 (−*q* of 30); mitochondrial reads were also excluded. Reads overlapping ENCODE mm10 blacklisted regions (v2) were subsequently removed using BEDTools (*112*) intersect (-v). PCR duplicates were identified and removed using the SAMtools fixmate and markdup pipeline. To correct for the 9-bp staggered insertion introduced by Tn5 transposase nick repair, reads were shifted +4 bp on the forward strand and −5 bp on the reverse strand using the alignmentSieve –ATACshift function from deepTools (*113*). Chromatin accessibility peaks were called on the deduplicated BAM files using MACS2 (*110*) (v2.2.7.1) in paired-end mode (-f BAMPE -g mm -q 0.01 –call-summits –keep-dup all); the –keep-dup all flag was used because duplicates had already been removed in the preceding step. Counts per million (CPM)–normalized bigwig files for genome browser visualization were generated from Tn5-shifted BAM files using bamCoverage from deepTools (--normalizeUsing CPM –effectiveGenomeSize 2308125349 –binSize 10 –extendReads –blackListFileName mm10-blacklist.v2.bed). Differential accessibility analysis was done using MACS2 (*110*), PCA was performed using DiffBind (v3.8.4), and peak annotation was performed using the R package ChIPseeker (*109*) (v3.15).

### DiffTF analysis on ATAC-seq

DiffTF (*42*) analysis was performed as previously published (*114*) in basic mode using the January 2012 GRCm38 mm10 target genome and motifs from the JASPAR library with default parameters (*P* value < 0.00001 and Bg base compositions of 0.29, 0.21, 0.21, and 0.29). Identified TFs were classified as significant if they had an FDR lower than 0.001 in at least one of the pairwise comparisons between sgCt and sg*Pbrm1*.

### ChIP-seq/ChIP-qPCR

NMuMG cells were seeded in 15-cm tissue culture plates with or without TGFβ1 as described above, such that they were ∼80 to 90% confluent on the day of harvesting (30 to 40 million cells/15 cm at harvest). Cells were either cross-linked with 1% formaldehyde only (histone modifications, Fosb, and Fosl2 ChIP-seq) for 10 min at RT or sequentially dual cross-linked (Brg1, Phf10, and Atf3 ChIP-seq) with 2 mM disuccinimidyl glutarate (DSG) for 45 min at RT followed by 1% formaldehyde for 10 min at RT as described in (*39*). Cross-linking was quenched with 125 mM glycine for 10 min at RT. The cells were washed with ice-cold 1× PBS, scraped from a plate, and flash frozen to be stored at −80°C until ready for processing. Cell pellets were thawed on ice, extracted with nuclear extraction buffer [50 mM Hepes-KOH (pH 8.0), 140 mM NaCl, 1 mM EDTA, 10% glycerol, 0.5% NP-40, and 0.25% Triton X-100] for 10 min on ice and washed once with nuclear wash buffer [10 mM tris-HCl (pH 8.0), 1 mM EDTA, 0.5 mM EGTA, and 200 mM NaCl]. Nuclear pellets were resuspended in shearing buffer [10 mM tris-HCl (pH 8.0), 1 mM EDTA, and 0.1% SDS] and sonicated using Branson SFX250 at 20% amplitude for 9 min for dual cross-linked cells and 6 min for single cross-linked cells at 0.5 s ON and 1.5 s OFF setting to obtain 200- to 800-bp fragment size. Sonicated chromatin was clarified by high-speed centrifugation, and a 50-μl aliquot was reverse cross-linked to check shearing efficiency. The chromatin was quantitated using absorbance at 260 nm and further diluted with 2× ChIP dilution buffer [100 mM Hepes-KOH (pH 7.5), 600 mM NaCl, 2 mM EDTA, 2% Triton X-100, 0.2% sodium deoxycholate, and 0.2% SDS] to obtain equal chromatin amounts in equal volume. Antibodies against BRG1 (ab110641, Abcam), PHF10 (PA5-30678, Invitrogen), ATF3 (ab207434, Abcam), H3K14ac (07-353, MilliporeSigma), H3K18ac (ab1191, Abcam), H3K27ac (ab4729, Abcam), H3K4me (ab8895, Abcam), H3K4me3 (ab8580, Abcam), Fosb (MA5-15056, Invitrogen), and Fosl2 (sc-166102, Santa Cruz) were added. After overnight primary antibody incubation at 4°C with rotation, Protein A (for rabbit antibodies) or Protein G (for mouse antibodies) Dynabeads were added and mixed using rotation for 2 hours at 4°C. The beads were sequentially washed with low-salt wash buffer [20 mM Hepes-KOH (pH 7.5), 0.1% SDS, 0.1% deoxycholate, 1% Triton X-100, 150 mM NaCl, 1 mM EDTA, and 0.5 mM EGTA], high-salt wash buffer [20 mM Hepes-KOH (pH 7.5), 0.1% SDS, 0.1% deoxycholate, 1% Triton X-100, 500 mM NaCl, 1 mM EDTA, and 0.5 mM EGTA], LiCl wash buffer [20 mM Hepes-KOH (pH 7.5), 0.5% deoxycholate, 0.5% IGEPAL CA-630 (octylphenyl-polyethylene glycol), 250 mM LiCl, 1 mM EDTA, 0.5 mM and EGTA], and final wash buffer [20 mM Hepes-KOH (pH 7.5), 1 mM EDTA, and 0.5 mM EGTA]. The immunoprecipitated chromatin was eluted once with 200 μl and once with 100 μl of elution buffer (100 mM NaHCO_3_ and 1% SDS) for 30 min each at 37°C with shaking. The eluate and the saved input were treated with 2 μl of RNase A (10 mg/ml; Thermo Fisher Scientific, EN0531) and 2 μl of Proteinase K (20 mg/ml; Thermo Fisher Scientific, EO0491) and reverse cross-linked at 65°C for 16 hours. DNA was extracted once with phenol:chloroform and once with chloroform and then precipitated by adding 1/10 volume of 3 M NaOAc (pH 5.2) and 1 volume 2-propanol and 2 μl of glycogen (20 mg/ml, Thermo Fisher Scientific, R0561) overnight at −20°C. After centrifugation at top speed (∼21,000*g*) for 1 hour, the pellet was washed with fresh 70% ethanol and then air-dried. DNA was resuspended in low-EDTA TE (Tris-EDTA) buffer (10 mM Tris, 0.1 mM EDTA pH 8.0), and DNA quality and concentration were determined using Qubit and Agilent TapeStation by Purdue Genomics Core Facility. For ChIP-qPCR, 4 μl of ChIPDNA was used per sample. ChIP-seq library preparation was done using the Ovation Ultralow System V2 UDI (Tecan Genomics) according to the manufacturer’s directions, and the final library was subjected to double-sided size selection of 0.65× and 1×. Library quality and concentration were determined using Qubit and Agilent TapeStation by Purdue Genomics Core Facility and submitted for 150-bp PE sequencing on a NovaSeq 6000 platform (Novogene, Sacramento, CA). Primers for ChIP-qPCR were as follows: mAtf3 FP: CCTTATCAGGCTGGGAGCCG; mAtf3 RP: GGTGGAGTCATGCCGCTG.

### ChIP-seq data processing

Quality trimming (adapter removal, phred quality cutoff ≥ Q30, and minimum read length of 50 bp after quality filtering) of raw reads was performed using the fastp (v0.19.5) tool. Trimmed reads were mapped to mouse mm10 genome build using the bowtie2 (*108*) (v2.3.3) aligner. BAM to BigWig conversion was performed using deeptools (*113*) (v3.5.1) with CPM normalization. Peak calling was performed with MACS3 (*110*) (v3.0.0a7) within Partek Flow (v11.0.24.0325) with -BROAD ON for SWI/SNF subunits. Peak annotation was performed using the R package ChIPseeker (*109*) (v3.15). Peak overlaps were performed using BEDtools (*112*) (v2.31.1), and heatmaps and metagene analyses were performed using deeptools (*113*) (v3.5.4).

### CUT&RUN

CUT&RUN was done according to the protocol available from EpiCypher (v2.2) with minor modifications ; in-house prepared pAG-MNase (protein A/G-Micrococcal Nuclease) was used, and incubation of cells with pAG-MNase was done at 4°C for 1 hour. To purify the pAG-MNase, the construct was obtained from Addgene (pAG/MNase was a gift from S. Henikoff; Addgene, plasmid #123461), and purification was done as described here (*115*). The amount of pAG-MNase used was 37 ng per reaction. Antibodies used were PHF10 (Invitrogen, #PA5-30678), H3K4me3 (EpiCypher, 13-0060), PBRM1 (Bethyl, #A301-591A), and rabbit control IgG (EpiCypher, #13-0042). Libraries were prepared using the CUTANA CUT&RUN Library Prep Kit (14-1001 and 14-1002) according to the manufacturer’s instructions, and the quality and concentration were determined using Qubit and Agilent TapeStation by Purdue Genomics Core Facility. Libraries were sequenced using 150-bp PE sequencing on an Aviti platform (Element Biosciences) by the Purdue Genomics Core Facility. All experiments were performed with two biological replicates.

### CUT&RUN data analysis

Raw paired-end reads were aligned using a dual-mapping strategy to separately quantify reads from the target genome and the exogenous *Escherichia coli* spike-in. Alignment to the mouse mm10 target genome was performed with Bowtie2 (*108*) using local alignment parameters appropriate for the short, size-selected CUT&RUN fragments (--local –very-sensitive-local –no-unal –no-mixed –no-discordant –phred33 -I 10 -X 700), which permit minor adapter read-through common in short fragments while maintaining alignment stringency. Alignment to the *E. coli* K12 MG1655 spike-in genome was performed in a separate run using end-to-end parameters (--end-to-end –very-sensitive –no-overlap –no-dovetail –no-unal –no-mixed –no-discordant –phred33 -I 10 -X 700) to avoid soft-clipping artifacts on the small bacterial reference. Resulting BAM files were filtered with SAMtools (*106*) to retain only properly paired, uniquely mapped reads (-f 2 -q 10). PCR duplicates were flagged using SAMtools markdup to assess library complexity and amplification efficiency but were not removed from downstream analysis. Reads overlapping ENCODE mm10 blacklisted regions (v2) were excluded using BEDTools (*112*) intersect (-v). A spike-in scaling factor was calculated for each sample as 100,000 divided by the number of high-quality, properly paired *E. coli* read pairs, providing cross-sample normalization that is independent of sequencing depth. Spike-in normalized bigwig tracks were generated using bamCoverage from deepTools (*113*) (--normalizeUsing None --scaleFactor [sample-specific value] --extendReads --exactScaling --binSize 10) for genome browser visualization. Chromatin occupancy peaks were called using MACS2 (*110*) in paired-end mode (-f BAMPE -q 0.05 --keep-dup all) against two independent IgG negative controls, one derived from sg*Pbrm1* cells and one from sgCt cells, to stringently account for background signal in both experimental backgrounds. Peak annotation was performed using the R package ChIPseeker (*109*) (v3.15).

### Expression and purification of human PBRM1 BDs

Constructs encoding codon-optimized open reading frames for bacterial expression of human PBRM1 BD2 (Addgene, #39013), BD3 (Addgene, #39030), BD4 (Addgene, #39103), BD5 (Addgene, #38999), and tandem BD2-5 (Structural Genomics Consortium, construct ID #PB1A-c080) were transformed in BL21 (DE3) for BDs and BL21 Rosetta2 (DE3) pLysS for tandem BD2-5. Protein was induced with 0.1 mM isopropyl-β-D-thiogalactopyranoside and expressed at 18°C overnight. Cultures were resuspended in binding buffer [20 mM tris (pH 8.0), 150 mM NaCl, 25 mM imidazole, and 5% glycerol with EDTA-free protease inhibitors], passed through a 18½ gauge needle 10 to 12 times, sonicated at 10% amplitude with 30 s ON 30 s OFF cycle for a total of 6 min, and centrifuged at 21,000*g* for 1 hour at 4°C. An aliquot of the supernatant was removed for the gel, and the rest of the supernatant was incubated with binding buffer–equilibrated Ni^+2^-NTA (Nickel-Nitrilotriacetic Acid) resin (HisPur Ni-NTA, #88221, Thermo Fisher Scientific) at 4°C for 2 hours with end-to-end rotation. The protein-bound resin was washed three times with wash buffer [20 mM tris (pH 8.0), 150 mM NaCl, 50 mM imidazole, and 5% glycerol with EDTA-free protease inhibitors] and eluted four times with elution buffer [20 mM tris (pH 8.0), 150 mM NaCl, and 5% glycerol with EDTA-free protease inhibitors] containing 100, 200, 300, and then 400 mM imidazole. The eluted proteins were checked using SDS–polyacrylamide gel electrophoresis, and pure elutions were combined and passed through Zeba spin desalting 7K molecular weight cutoff (MWCO) columns (#89892, Thermo Fisher Scientific). Purified proteins were stored in storage buffer [20 mM tris (pH 8.0), 150 mM NaCl, and 20% glycerol] at −80°C. These proteins were sent to Epicypher for further binding analysis.

### Captify peptide and nucleosome binding assays

The assay previously known as dCypher is now named Captify, with no distinction in how the assay is performed or its capabilities. Assays were performed as described previously with minor modifications (*53*, *116*–*118*). Briefly, 5 μl of the target (biotinylated histone peptides, 100 nM; or biotinylated nucleosomes, 10 nM; EpiCypher) was incubated with 5 μl of Query for 30 min at 23°C. Interactions were detected by sequential addition of 5 μl of Nickel Chelate Acceptor beads (20 μg/ml, 30 min at 23°C) followed by 5 μl of streptavidin donor beads (40 μg/ml, 30 min at 23°C in the dark) before measurement of Alpha Counts on a 2104 EnVision Plate Reader (Revvity).

This study uses a nomenclature recently devised for accurate scientific communication in the chromatin and epigenetic fields (*119*). Here, ([H3K4me3]_2_) indicates a fully defined semisynthetic homotypic nucleosome where other positions not denoted are understood to be definitively unmodified on major histones. For peptide binding curves, Queries were titrated in an optimized peptide binding buffer [50 mM tris (pH 7.5), 25 mM NaCl, 0.01% Tween 20, and 0.01% BSA]. For nucleosome assays, Queries were first cross-titrated against salt conditions (25 to 200 mM NaCl in 25 mM increments) in base nucleosome binding buffer [20 mM tris (pH 7.5), 0.01% NP-40, 1 mM dithiothreitol, and 0.01% BSA] using ([H3.1]_2_) unmodified, ([H3.1K14ac]_2_), ([H3.1K4acK9acK14acK18ac]_2_), and ([H3.1K4me3K9acK14acK18ac]_2_) nucleosomes to determine optimal probing conditions for a nucleosome discovery screen. Optimal binding conditions were based on the relative EC_50_ (EC_50_^rel^) values for H3K14ac nucleosome binding for each Query: 500 nM BD2 and 25 mM NaCl; 107 nM BD3 and 125 mM NaCl; 316 nM BD4 and 75 mM NaCl; 1000 nM BD5 and 25 mM NaCl; and 33 nM tandem BD2-5 and 150 mM NaCl. These optimized binding conditions were then used to determine EC_50_^rel^ values for each BD against a panel of modified nucleosomes selected from discovery screen hits. Data were analyzed using four-paremeter logistic nonlinear regression in GraphPad Prism 10.

### Patient data

Comparison of PBRM1 mRNA expression between normal breast tissue and tumor tissue was performed in GEPIA (Gene Expression Profiling Interactive Analysis) using TCGA data. Kaplan-Meier plots were generated using the Km plotter (*120*).

### ChIP-seq data processing of published datasets

Dpf2 and Pbrm1 ChIP-seq datasets from GSE249211 (*38*) were imported into Partek Flow (v11.0.24.0325). Reads were aligned to mouse genome mm10 build using Bowtie2 (*108*) (v2.2.5) allowing one mismatch. Alignments were filtered for MAPQ > 10 using Partek Flow Filter alignments (v11.0.24.0325). Filtered BAMs were used for bigwig generation using deeptools (*113*) bamcoverage with --normalizeUsing CPM -p 32 –effectiveGenomeSize 2308125299 --extendReads. Peak calling was done using MACS (*110*) (v3.0.0a7) in Partek Flow (v11.0.24.0325) with default parameters and -broad ON mode. Irf1 ChIP-seq datasets from GSE141501 (*49*) were imported into Partek Flow (v10.0.22.1111), reads were trimmed for a quality of >20 and a minimum length of >20 using Trim bases in Partek Flow (v10.0.22.1111). Trimmed reads were aligned to mouse genome mm10 build using Bowtie2 (v2.2.5) allowing one mismatch. BAMs were used for bigwig generation using deeptools bamcoverage with --normalizeUsing CPM -p 32 --effectiveGenomeSize 2494787188. Peak calling was done using MACS (v3.0.0a7) in Partek Flow (v10.0.22.1111) with default parameters. Snail and slug ChIP-seq datasets from GSE61198 (*50*) were imported into Partek Flow (v12.6.0). Reads were aligned to mouse genome mm10 build using Bowtie (v1.0.0) with default parameters. Alignments were filtered for MAPQ > 10 and blacklisted regions using SAMtools (*106*) and BEDtools (*112*). Filtered BAMs were used for bigwig generation using deeptools bamcoverage with --normalizeUsing CPM -p 32 --effectiveGenomeSize 2308125299 --extendReads 200 parameters.
