# Supplementary material for: PBRM1-dependent PBAF targeting is required for EMT and metastasis in breast cancer
Source: Sci Adv. 2026 Jul 29;12(31):eaed8038. doi: 10.1126/sciadv.aed8038 (PMC13418929; doi:10.1126/sciadv.aed8038)
Supplement: Supplementary file 1 — Figs. S1 to S7 Legends for tables S1 to S3 [file sciadv.aed8038_sm.pdf]

Science Advances
AAAS

Supplementary Materials for

PBRM1-dependent PBAF targeting is required for EMT and metastasis in breast cancer

Alisha Dhiman *et al.*

Corresponding author: Emily C. Dykhuizen, edykhui@purdue.edu

Sci. Adv. **12**, eaed8038 (2026)
DOI: 10.1126/sciadv.aed8038

The PDF file includes:

Figs. S1 to S7
Legends for tables S1 to S3

Other Supplementary Material for this manuscript includes the following:

Tables S1 to S3

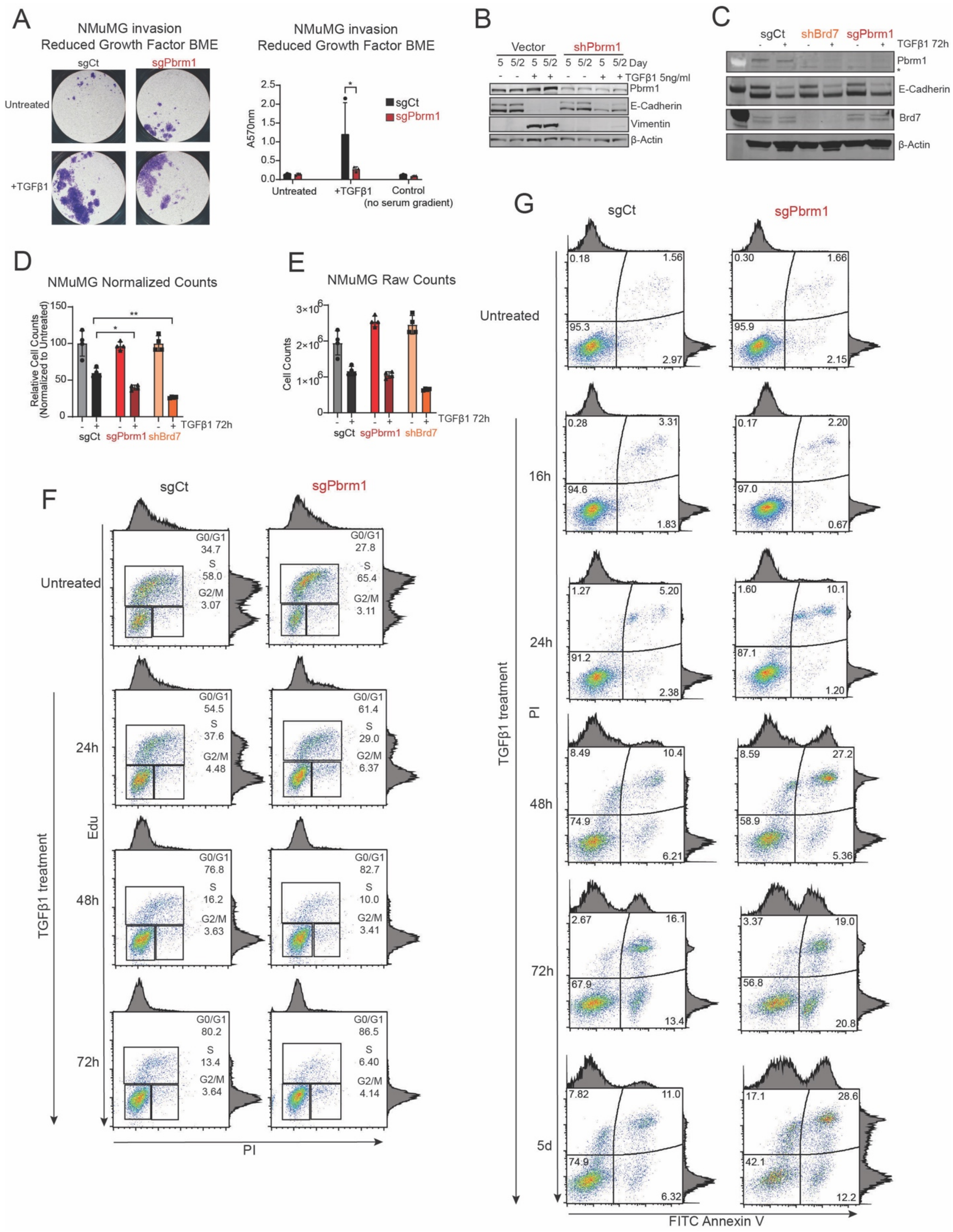
A
NMuMG invasion
Reduced Growth Factor BME
sgCt
sgPbrm1
Untreated
+TGFβ1
NMuMG invasion
Reduced Growth Factor BME
A570nm
sgCt
sgPbrm1
Untreated
+TGFβ1
Control
(no serum gradient)
B
Vector
shPbrm1
Day
TGFβ1 5ng/ml
Pbrm1
E-Cadherin
Vimentin
β-Actin
C
sgCt
shBrd7
sgPbrm1
TGFβ1 72h
Pbrm1
E-Cadherin
Brd7
β-Actin
D
NMuMG Normalized Counts
Relative Cell Counts
(Normalized to Untreated)
TGFβ1 72h
sgCt
sgPbrm1
shBrd7
E
NMuMG Raw Counts
Cell Counts
TGFβ1 72h
sgCt
sgPbrm1
shBrd7
F
sgCt
sgPbrm1
Untreated
24h
48h
72h
TGFβ1 treatment
Edu
PI
G
sgCt
sgPbrm1
Untreated
16h
24h
48h
72h
5d
TGFβ1 treatment
PI
FITC Annexin V

SI Figure 1:

(A) Transwell invasion assay images and bar plot of absorbance quantification of NMuMG sgCt and sg*Pbrm1* cells with and without TGFβ1 treatment in reduced growth factor basement membrane (BME). n=3 technical replicates. Data are represented as mean ± SD.

(B) Immunoblots of Pbrm1, E-Cadherin, and Vimentin in NMuMG control and sh*Pbrm1* cells with and without TGFβ1 treatment with the indicated concentration and time periods. 5/2 denotes 5 days of TGFβ1 treatment followed by 2 days without TGFβ1.

(C) Immunoblots of Pbrm1 and E-Cadherin levels in NMuMG sgCt, sh*Brd7,* and sg*Pbrm1* cells with and without TGFβ1 treatment using whole cell extracts.

(D and E) Relative (D) and absolute (E) cell counts of NMuMG sgCt, sg*Pbrm1,* and sh*Brd7* cells with and without TGFβ1 treatment, normalized to untreated sample for relative counts. Representative graph, n=2 biological replicates. Data are represented as mean ± SD.

(F) Flow cytometry density dot plots of Edu-PI staining of percentage of cells in different cell cycle stages in NMuMG sgCt and sg*Pbrm1* cells with and without TGFβ1 treatment for the indicated time periods. n=3 biological replicates. Data are represented as mean ± SD.

(G) Flow cytometry density dot plots of AnnexinV-PI staining of percentage of live (AnnexinV- PI-, Q4), Annexin V+ (Q2 and Q3) and PI+ (Q1) cells in NMuMG sgCt and sg*Pbrm1* cells with and without TGFβ1 treatment for the indicated time periods. n=2 biological replicates. Data are represented as mean ± SD.

Statistical comparison was done using multiple unpaired t-tests with Holm-Sidak correction. ✱: $p < 0.05$, ✱✱: $p < 0.01$, ✱✱✱: $p < 0.001$, ✱✱✱✱: $p < 0.0001$

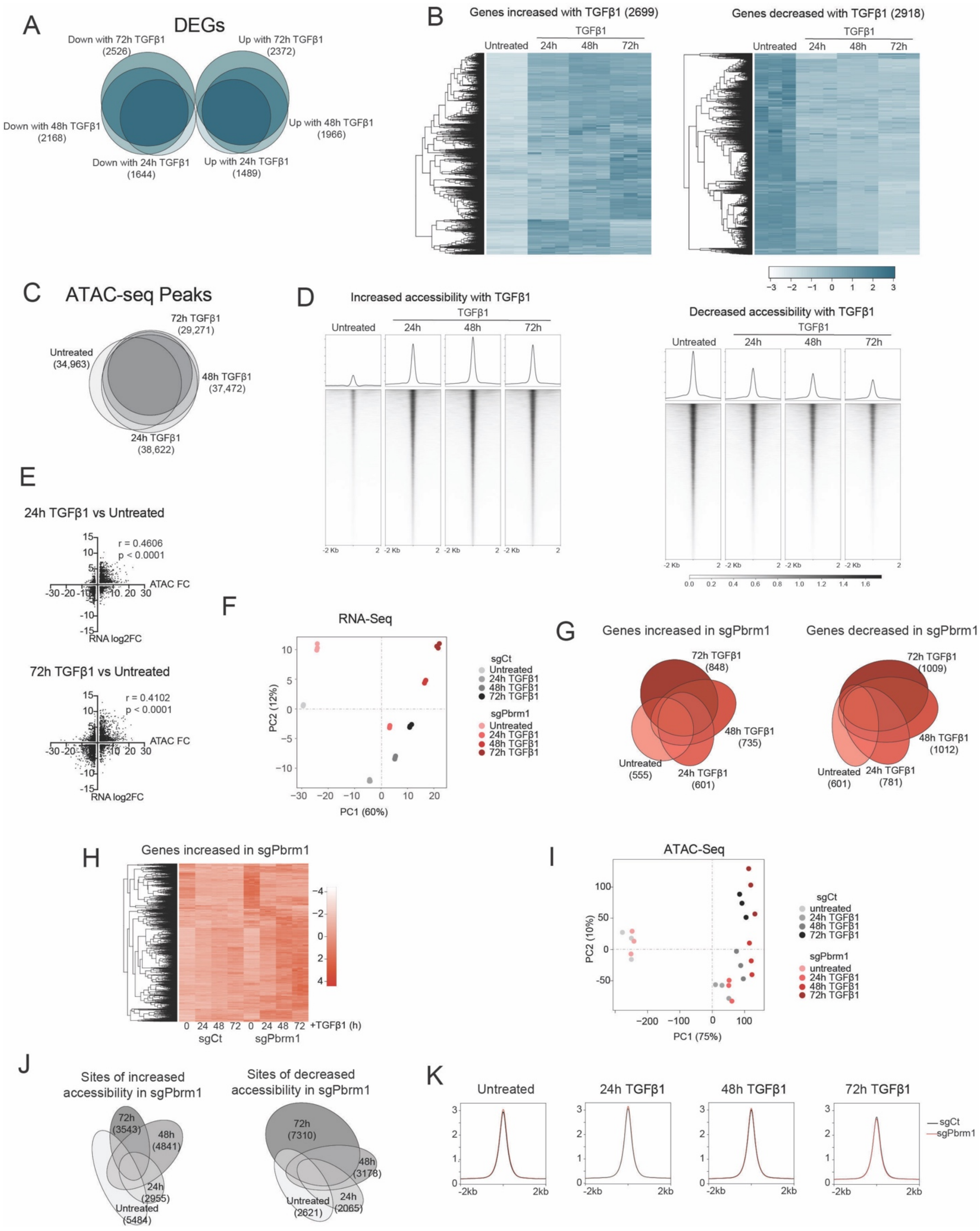

SI Figure 2

(A) Overlap of DEGs shared between the indicated conditions, as identified in RNA-seq. Total number of DEGs in each condition is indicated in parentheses.

(B) Heatmap representation of genes increased (left) or decreased (right) in NMuMG sgCt cells with TGFβ1 treatment at different time points.

(C) Overlap of accessible regions, as identified in ATAC-seq. Total number of regions in each condition is indicated in parentheses.

(D) Metagene plots and heatmaps of regions of differential increased (left) or decreased (right) accessibility in NMuMG sgCt cells with TGFβ1 treatment. The set of regions includes sites with differential accessibility at any time point of TGFβ1 treatment compared to untreated cells.

(E) Scatter plot of differentially accessible regions from ATAC-seq with differential expression of the nearest gene from RNA-Seq, at 24h (top) and 72h (bottom) of TGFβ1 treatment compared to untreated NMuMG sgCt cells. Each data point in the scatter plot represents on the *x axis*: differentially accessible regions as FC from ATAC-seq and on the *y axis*: expression change of the nearest gene from RNA-seq as log2FC with 24 or 72h TGFβ1 treatment relative to no treatment.

(F) Principal Component Analysis of the RNA-seq data of NMuMG sgCt and sg*Pbrm1* cells with and without TGFβ1 treatment for the designated times.

(G) Overlap of DEGs shared between the indicated conditions, as identified in RNA-seq. Total number of DEGs in each condition is indicated in parentheses.

(H) Heatmap representation of all genes increased in NMuMG sg*Pbrm1* relative to sgCt cells at any timepoint of TGFβ1 treatment compared to untreated cells.

(I) Principal Component Analysis of the ATAC-seq data for NMuMG sgCt and sg*Pbrm1* cells with and without TGFβ1 treatment where samples are color coded by treatment.

(J) Overlapof differentially accessible regions (increased (left) or decreased (right)) shared between the indicated conditions, as identified in ATAC-seq. Total number of regions in each condition is indicated in parentheses.

(K) Metagene plots of global accessibility in NMuMG sgCt and sg*Pbrm1* cells in untreated and TGFβ1 treated conditions as identified in ATAC-seq. The regions used for the average peak size are the overlap of all peaks identified from any condition/genotype.

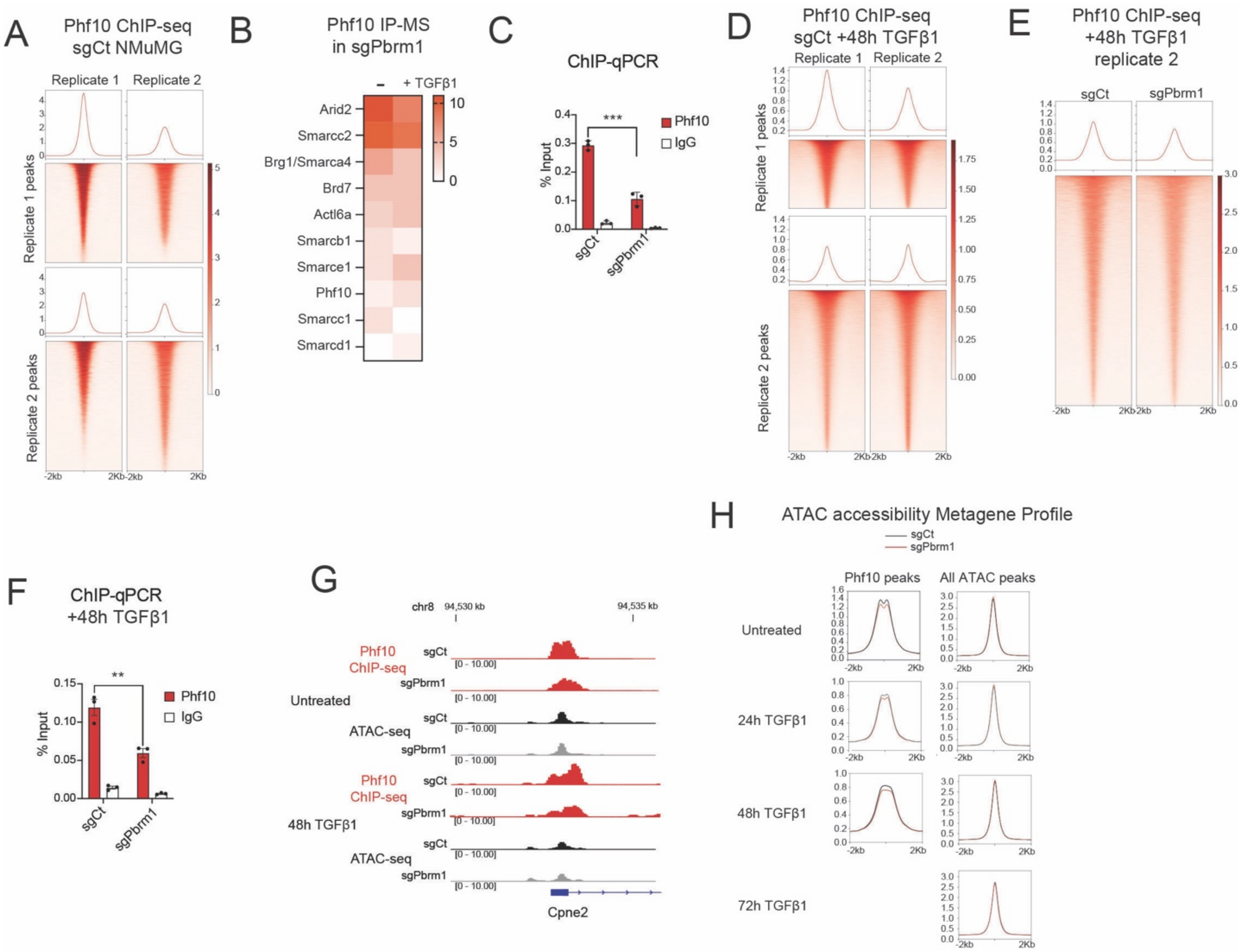

SI Figure 3

(A) Metagene plots and heatmaps of ChIP-seq enrichment of Phf10 at Phf10 sites identified in two independent ChIP-seq replicates in untreated NMuMG sgCt cells.

(B) Heatmap representation of PBAF subunit peptide abundance in NMuMG sg*Pbrm1* cells with and without TGFβ1 treatment identified by Phf10 IP-MS.

(C) Bar plot of the percent of input DNA enriched from ChIP of Phf10 and IgG in NMuMG sgCt and sg*Pbrm1* cells using qPCR at ATF3 locus. N = 3 independent replicates.

(D) Metagene plots and heatmaps of ChIP-seq enrichment of Phf10 at Phf10 sites identified in two independent ChIP-seq replicates in 48h TGFβ1 treated NMuMG sgCt cells.

(E) Metagene plots and heatmaps of ChIP-seq enrichment of Phf10 in 48h TGFβ1 treated NMuMG sgCt and sg*Pbrm1* cells using Phf10 peaks identified in NMuMG sgCt cells with 48h TGFβ1.

(F) Bar plot of the percent of input DNA enriched from ChIP of Phf10 and IgG in NMuMG sgCt and sg*Pbrm1* cells with 48h TGFβ1 treatment using qPCR at ATF3 locus. N = 3 independent replicates.

(G) Genomic tracks of ChIP-seq enrichment of Phf10 and ATAC-seq profile in untreated and 48h TGFβ1-treated NMuMG sgCt and sg*Pbrm1* cells at *Cpne2* locus

(H) Metagene plots of accessibility profile of NMuMG sgCt and sg*Pbrm1* cells with 0-72h TGFβ1 treatment at all regions identified in ATAC-seq, compared to accessibility profile at Phf10 ChIP-seq peaks identified in NMuMG sgCt cells with 0, 24 or 48h TGFβ1 treatment.

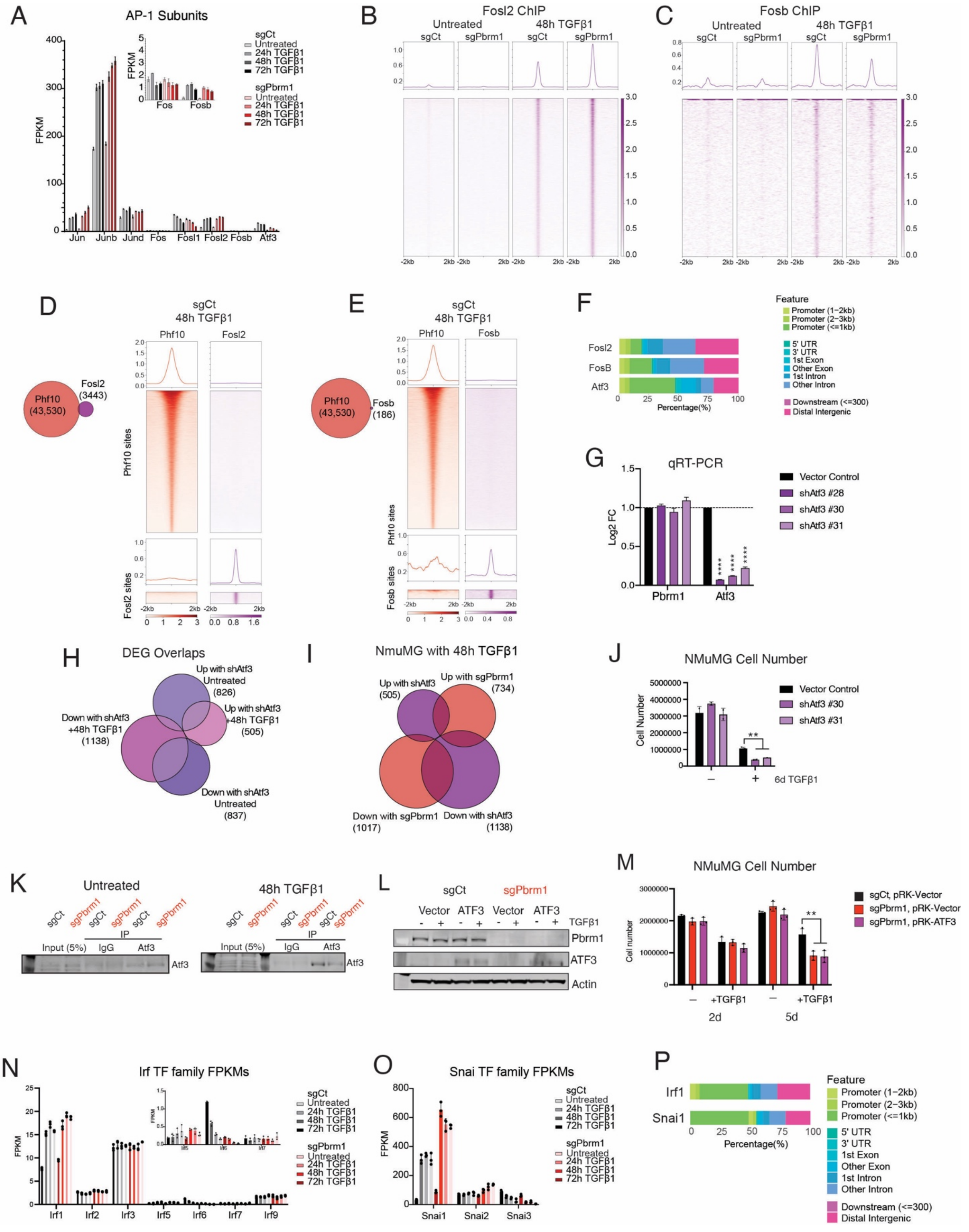
A
AP-1 Subunits
sgCt
Untreated
24h TGFβ1
48h TGFβ1
72h TGFβ1
sgPbrm1
Untreated
24h TGFβ1
48h TGFβ1
72h TGFβ1
FPKM
Jun Junb Jund Fos Fosl1 Fosl2 Fosb Atf3
B
Fosl2 ChIP
Untreated
48h TGFβ1
sgCt sgPbrm1 sgCt sgPbrm1
C
Fosb ChIP
Untreated
48h TGFβ1
sgCt sgPbrm1 sgCt sgPbrm1
D
sgCt
48h TGFβ1
Phf10 Fosl2
Phf10 (43,530)
Fosl2 (3443)
Phf10 sites
Fosl2 sites
E
sgCt
48h TGFβ1
Phf10 Fosb
Phf10 (43,530)
Fosb (186)
Phf10 sites
Fosb sites
F
Feature
Promoter (1−2kb)
Promoter (2−3kb)
Promoter (<=1kb)
5' UTR
3' UTR
1st Exon
Other Exon
1st Intron
Other Intron
Downstream (<=300)
Distal Intergenic
Fosl2
FosB
Atf3
Percentage(%)
G
qRT-PCR
Vector Control
shAtf3 #28
shAtf3 #30
shAtf3 #31
Log2 FC
Pbrm1 Atf3
H
DEG Overlaps
Up with shAtf3 Untreated (826)
Up with shAtf3 +48h TGFβ1 (505)
Down with shAtf3 +48h TGFβ1 (1138)
Down with shAtf3 Untreated (837)
I
NmuMG with 48h TGFβ1
Up with shAtf3 (505)
Up with sgPbrm1 (734)
Down with sgPbrm1 (1017)
Down with shAtf3 (1138)
J
NMuMG Cell Number
Vector Control
shAtf3 #30
shAtf3 #31
Cell Number
6d TGFβ1
K
Untreated
48h TGFβ1
sgCt sgPbrm1 sgCt sgPbrm1 sgCt sgPbrm1
Input (5%) IgG Atf3
IP
Atf3
L
sgCt sgPbrm1
Vector ATF3 Vector ATF3
TGFβ1
Pbrm1
ATF3
Actin
M
NMuMG Cell Number
sgCt, pRK-Vector
sgPbrm1, pRK-Vector
sgPbrm1, pRK-ATF3
Cell number
+TGFβ1
2d 5d
N
Irf TF family FPKMs
sgCt
Untreated
24h TGFβ1
48h TGFβ1
72h TGFβ1
sgPbrm1
Untreated
24h TGFβ1
48h TGFβ1
72h TGFβ1
FPKM
Irf1 Irf2 Irf3 Irf5 Irf6 Irf7 Irf9
O
Snai TF family FPKMs
FPKM
Snai1 Snai2 Snai3
P
Feature
Irf1
Snai1
Percentage(%)

SI Figure 4

(A) Bar plot of FPKM values for the different AP-1 subunits from RNA-seq of NMuMG sgCt and sg*Pbrm1* cells with 0, 24, 48, and 72h TGFβ1 treatment.

(B) Metagene plots and heatmaps of ChIP-seq enrichment of Fosl2 in sgCt and sg*Pbrm1* cells in both untreated and 48h TGFβ1-treated conditions. Enrichment was plotted for corresponding TF binding sites compiled from all conditions.

(C) Metagene plots and heatmaps of ChIP-seq enrichment of Fosb in sgCt and sg*Pbrm1* cells in both untreated and 48h TGFβ1-treated conditions. Enrichment was plotted for corresponding TF binding sites compiled from all conditions.

(D) Overlap of Phf10 ChIP-Seq sites with Fosl2 ChIP-seq sites in sgCt cells with 48h TGFβ1 treatment. Total number of peaks identified in ChIP-seq for each protein is indicated in parentheses. To the right of each Venn diagram are the metagene plots and heatmaps of ChIP-seq enrichment of Phf10 with Fosl2 at the Phf10 (top) and Fosl2 (bottom) binding sites.

(E) Overlap of Phf10 ChIP-Seq sites with Fosb ChIP-seq sites in sgCt cells with 48h TGFβ1 treatment. Total number of peaks identified in ChIP-seq for each protein is indicated in parentheses. To the right of each Venn diagram are the metagene plots and heatmaps of ChIP-seq enrichment of Phf10 with Fosb at the Phf10 (top) and Fosb (bottom) binding sites.

(F) Genomic feature distribution of the Fosl2, Fosb, and Atf3 ChIP-seq peaks.

(G) qRT-PCR of NMuMG cells treated with three different shRNA against *Atf3*. *Oaz1* was used as the housekeeping gene. Data are represented as mean ± SD.

(H) Overlap of DEGs from RNA-seq of sh*Atf3* relative to control cells in both untreated and 48h TGFβ1-treated cells. Total number of DEGs in each condition is indicated in parentheses.

(I) Overlap between DEGs in sg*Pbrm1* vs sgCt and DEGs from sh*Atf3* vs shScr, both with 48h TGFβ1 treatment. Total number of genes from each condition is indicated in parentheses.

(J) Absolute cell counts of NMuMG control and sh*Atf3* cells with and without TGFβ1 treatment. 0.9 million cells/well were seeded for each cell line and cell counts were taken on day 6. Representative graph, n=3 biological replicates. Data are represented as mean ± SD.

(K) Immunoblots of lysates and immunoprecipitations from NMuMG sgCt and sg*Pbrm1* cells in untreated and 48h TGFβ1 treated conditions. n=2 biological replicates.

(L) Immunoblots of whole cell extracts from NMuMG sgCt and sg*Pbrm1* cells with human ATF3 expression.

(M) Absolute cell counts of NMuMG sgCt, sg*Pbrm1* and sg*Pbrm1*-*ATF3* re-expressing cells with and without 5 ng/mL TGFβ1 treatment. 0.8 million cells/well were seeded for each cell line and cell counts were taken on day 2 and 5. Representative graph, n=4 biological replicates. Data are represented as mean ± SD.

(N and O) Bar plots of FPKM values for the Irf (N) and Snai (O) TFs from RNA-seq of NMuMG sgCt and sg*Pbrm1* cells with 0, 24, 48, and 72h TGFβ1 treatment.

(P) Genomic feature distribution of the ChIP-seq peaks identified for Irf1 in NMuMG cells with 48h TGFβ1, and Snai1 in pBI.3G cells.

Statistical comparison was done using multiple unpaired t-tests with Holm-Sidak correction. ✱: $p < 0.05$, ✱✱: $p < 0.01$, ✱✱✱: $p < 0.001$, ✱✱✱✱: $p < 0.0001$

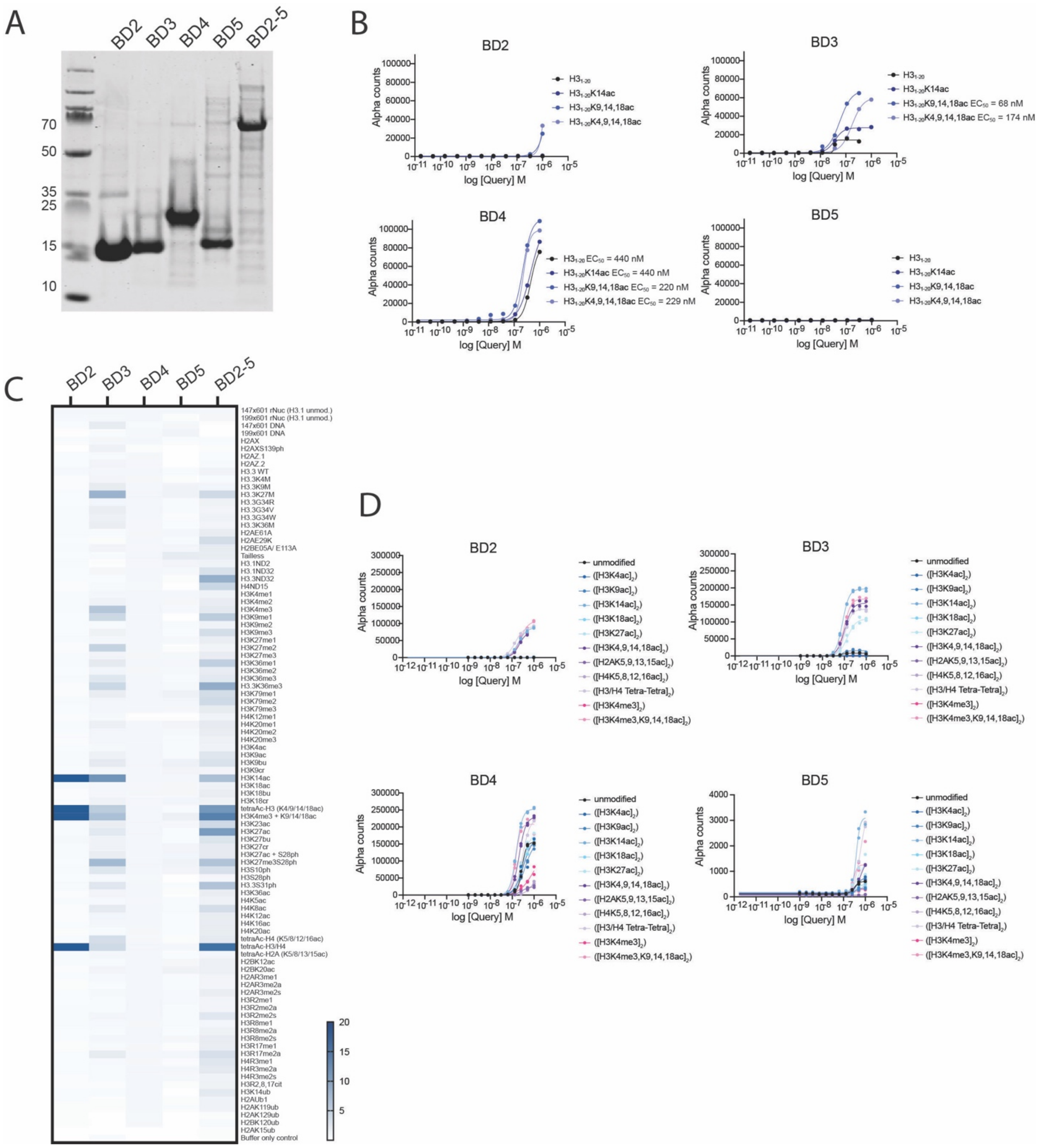

SI Figure 5

(A) Coomassie gel of the purified recombinant proteins BD2, BD3, BD4, BD5 and the tandem BD2-5 used for peptide and nucleosome binding assays.

(B) Binding curves of BD2, BD3, BD4, and BD5 with the indicated peptides obtained using the Captify™ assay. EC_{50} (nM) values are indicated in the legend.

(C) Heatmap representation of signal in the Captify™ assay with BD2, BD3, BD4, BD5, and tandem BD2-5 and nucleosomes with the indicated modifications. The data presented as Alpha Counts normalized to the signal from unmodified 147x601 rNucs.

(D) Binding curves of BD2, BD3, BD4 and BD5 for nucleosomes bearing the indicated peptides obtained using the Captify™ assay. EC_{50} (nM) values are listed in the table in Fig 5D.

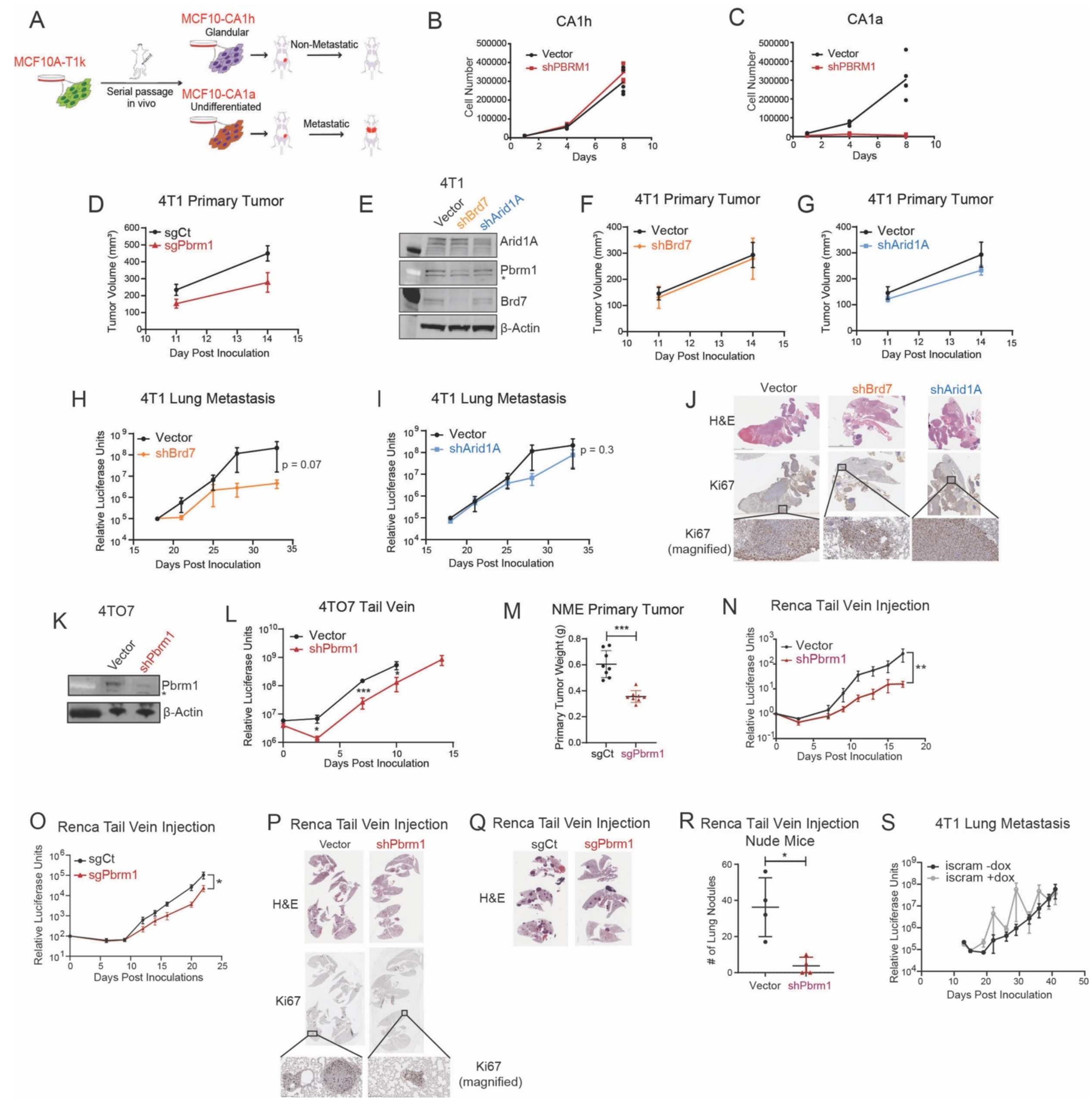

SI Figure 6

(A) Schematic diagram of the development of MCF10 series of cell lines- T1K, CA1h, and CA1a.

(B and C) *In vitro* proliferation of MCF10 CA1h (B) and CA1a (C) control and sh*Pbrm1* cells; 10,000 cells were plated at day 0. n=3 biological replicates. Two-way ANOVA was used for statistical comparison. Data are represented as mean ± SD.

(D) Primary tumor growth in 4T1 sgCt and sg*Pbrm1* cells measured using vernier calipers at the indicated time points. Two-way ANOVA with multiple comparisons was used for statistical comparison. Data are represented as mean ± SEM.

(E) Immunoblots of whole cell lysates from 4T1 cells expressing control vector, sh*Brd7* and sh*Arid1A.*

(F and G) Primary tumor growth in 4T1 sh*Brd7 (*F) and sh*Arid1A* (G) relative to control vector cells, measured using vernier calipers at the indicated time points. Two-way ANOVA with multiple comparisons was used for statistical comparison. Data are represented as mean ± SEM.

(H and I) Bioluminescent imaging of lung metastasis in 4T1 sh*Brd7* (H) and sh*Arid1A* (I) relative to vector control cells after removal of the primary tumor. Two-way ANOVA with multiple comparisons was used for statistical comparison. Data are represented as mean ± SEM.

(J) H&E and Ki67 stained IHC images of the lungs harvested at the end of the experiment described in (H) and (I).

(K) Immunoblots of whole cell lysates from 4TO7 cells expressing sh*Pbrm1* or vector control.

(L) Bioluminescent imaging of lung metastasis in 4TO7 vector control and sh*Pbrm1* cells. Welch's t-test was used for statistical comparison. Data are represented as mean ± SEM.

(M) Primary tumor weights from individual mice in the NME sgCt and sg*Pbrm1* groups harvested at the end of experiment. Welch's t-test was used for statistical comparison. Data are represented as mean ± SD.

(N and O) Bioluminescent imaging of lung metastasis in Renca sh*Pbrm1* (N) and sg*Pbrm1* (O) relative to control cells. Two-way ANOVA with multiple comparisons was used for statistical comparison. Data are represented as mean ± SEM.

(P) H&E and Ki67 stained IHC images of the lungs harvested at the end of the experiment described in (N).

(Q) H&E images of the lungs harvested at the end of the experiment described in (O).

(R) Number of lung nodules from individual mice in the Renca control and sh*Pbrm1* groups harvested at the end of experiment. Welch's t-test was used for statistical comparison. Data are represented as mean ± SD.

(S) Bioluminescent imaging of lung metastasis in 4T1 iscram cells with and without doxycycline administration, after removal of the primary tumor. Two-way ANOVA with multiple comparisons was used for statistical comparison. Data are represented as mean ± SEM.

✱: $p < 0.05$, ✱✱: $p < 0.01$, ✱✱✱: $p < 0.001$, ✱✱✱✱: $p < 0.0001$

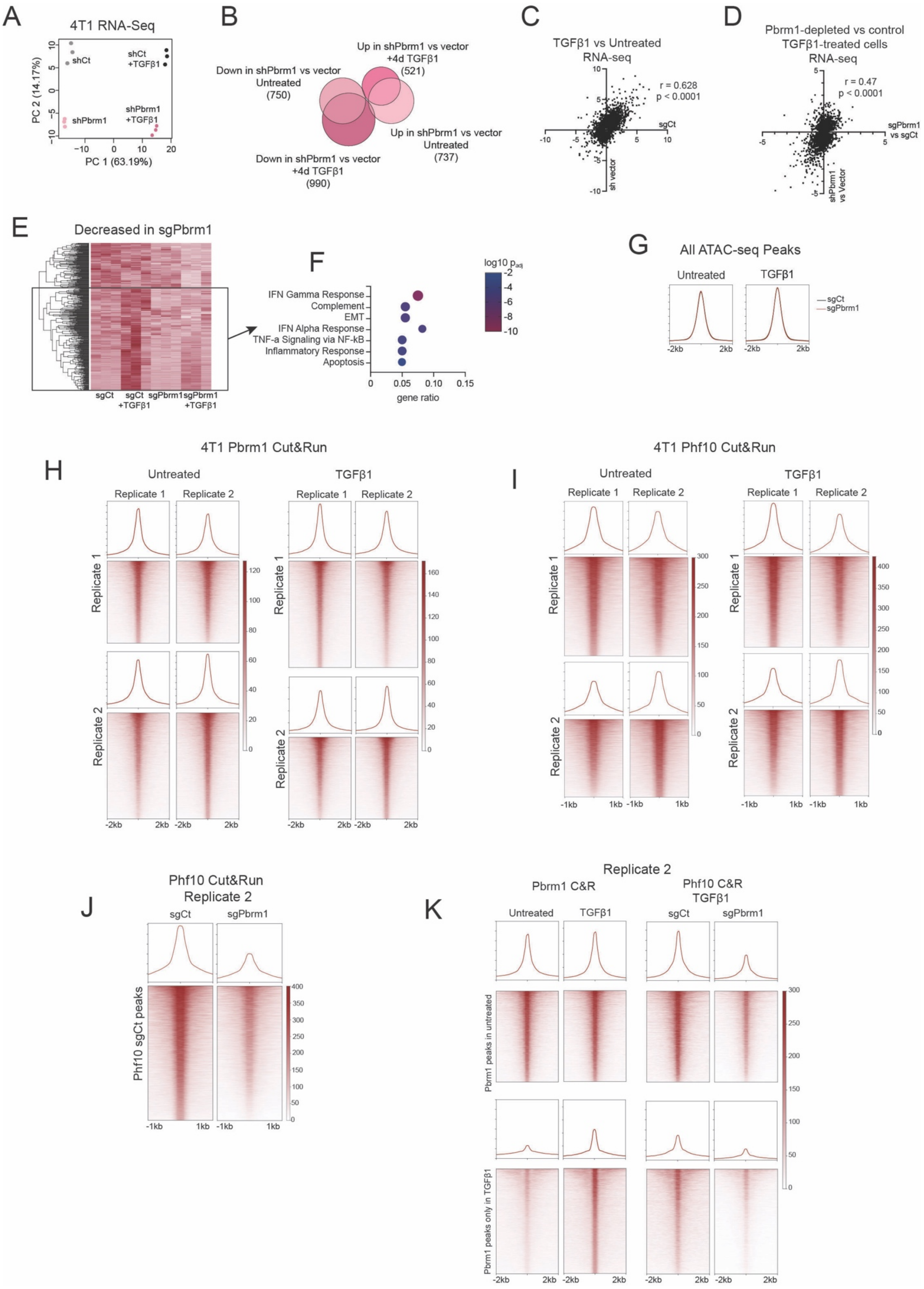
A
4T1 RNA-Seq
shCt
shCt +TGFβ1
shPbrm1
shPbrm1 +TGFβ1
PC 2 (14.17%)
PC 1 (63.19%)
B
Down in shPbrm1 vs vector Untreated (750)
Up in shPbrm1 vs vector +4d TGFβ1 (521)
Up in shPbrm1 vs vector Untreated (737)
Down in shPbrm1 vs vector +4d TGFβ1 (990)
C
TGFβ1 vs Untreated RNA-seq
r = 0.628
p < 0.0001
sgCt
sh vector
D
Pbrm1-depleted vs control TGFβ1-treated cells RNA-seq
r = 0.47
p < 0.0001
sgPbrm1 vs sgCt
shPbrm1 vs Vector
E
Decreased in sgPbrm1
sgCt
sgCt +TGFβ1
sgPbrm1
sgPbrm1 +TGFβ1
F
IFN Gamma Response
Complement
EMT
IFN Alpha Response
TNF-a Signaling via NF-kB
Inflammatory Response
Apoptosis
gene ratio
log10 p adj
G
All ATAC-seq Peaks
Untreated
TGFβ1
sgCt
sgPbrm1
-2kb
2kb
4T1 Pbrm1 Cut&Run
H
Untreated
TGFβ1
Replicate 1
Replicate 2
-2kb
2kb
4T1 Phf10 Cut&Run
I
Untreated
TGFβ1
Replicate 1
Replicate 2
-1kb
1kb
J
Phf10 Cut&Run Replicate 2
sgCt
sgPbrm1
Phf10 sgCt peaks
-1kb
1kb
K
Replicate 2
Pbrm1 C&R
Phf10 C&R TGFβ1
Untreated
TGFβ1
sgCt
sgPbrm1
Pbrm1 peaks in untreated
Pbrm1 peaks only in TGFβ1
-2kb
2kb

SI Figure 7

(A) Principal Component Analysis of the RNA-seq profile of 4T1 shCt and sh*Pbrm1* cells with and without TGFβ1 treatment where samples are color coded by treatment.

(B) Venn diagram of DEGs identified in RNA-seq of 4T1 cells with untreated sh*Pbrm1* relative to vector control cells and 4d TGFβ1 treated sh*Pbrm1* relative to vector control cells. Total number of DEGs in each condition is indicated in parentheses.

(C) Scatter plot of TGFβ1-induced gene expression changes in RNA-seq between 4T1 sgCt and shRNA vector control from SI 7B. Each data point in the scatter plot represents the log2FC expression value of a single gene in the indicated comparison, x axis: sgCt cells (TGFβ1 vs untreated), y axis: shRNA vector control cells (TGFβ1 vs untreated). The degree of correlation was calculated using all DEGs.

(D) Scatter plot of Pbrm1-depleted vs. control gene expression changes in TGFβ1-treated 4T1 cells from RNA-seq. Each data point in the scatter plot represents the log2FC expression value of a single gene in the indicated comparison, x axis: sgPbrm1 vs. sgCt cells, y axis: shPbrm1 vs. shRNA vector control cells. The degree of correlation was calculated using all DEGs.

(E) Heatmap representation of genes decreased in 4T1 sgPbrm1 relative to sgCt cells with and without TGFβ1 treatment. Genes induced by TGFβ1 in sgCt but not in sgPbrm1 cells are highlighted.

(F) Top overrepresented gene sets from pathway analysis using Enrichr on the highlighted subset of genes from (E).

(G) Metagene plots of global accessibility in 4T1 sgCt and sgPbrm1 cells in untreated and TGFβ1-treated conditions as identified in ATAC-seq. The regions used for the average peak size are the overlap of all peaks identified from any condition/genotype.

(H and I) Metagene plots and heatmaps of Pbrm1 CUT&RUN enrichment at Pbrm1 sites (H) and Phf10 CUT&RUN enrichment at Phf10 sites (I) as identified in two independent biological replicates in untreated and TGFβ1-treated 4T1 cells. Peak summits are aligned at the center.

(J) Metagene plots and heatmaps of Phf10 CUT&RUN enrichment in 4T1 sgCt and sgPbrm1 cells at Phf10 sgCt binding sites from biological replicate 2. Peak summits are aligned at the center.

(K) Metagene plots and heatmaps of Pbrm1 CUT&RUN enrichment in untreated and TGFβ1-treated 4T1 sgCt cells (left) and Phf10 CUT&RUN enrichment in sgCt and sgPbrm1 4T1 cells treated with TGFβ1 (right) from biological replicate 2. Enrichment was plotted at Pbrm1 sites from untreated cells (top) and Pbrm1 sites in TGFβ1-treated, cells (bottom). Peak summits are aligned at the center.

Additional Supplementary Material:

Excel spreadsheet with Antibodies, Reagents, Software (Tab 1), Oligos, Plasmids, Proteins (Tab 2), Bacterial strains, cell lines, mice strain (Tab 3).
